# Designing TiO_2_ Nanotubular Arrays with Au-CoO_*x*_ Core–Shell Nanoparticles for Enhanced Photoelectrochemical Methanol and Lignin Oxidation

**DOI:** 10.1021/acsami.4c07498

**Published:** 2024-09-04

**Authors:** Sabiha Sultana, Izabela Darowska, Marcin Pisarek, Grzegorz D. Sulka, Karolina Syrek

**Affiliations:** † Department of Physical Chemistry and Electrochemistry, Jagiellonian University, Gronostajowa 2, 30-387 Krakow, Poland; ‡ Laboratory of Surface Analysis, Institute of Physical Chemistry, Polish Academy of Sciences, Kasprzaka 44/52, 01-224 Warsaw, Poland

**Keywords:** nanotube array, photoelectrochemical oxidation, methanol, lignin, anodic TiO_2_, core−shell

## Abstract

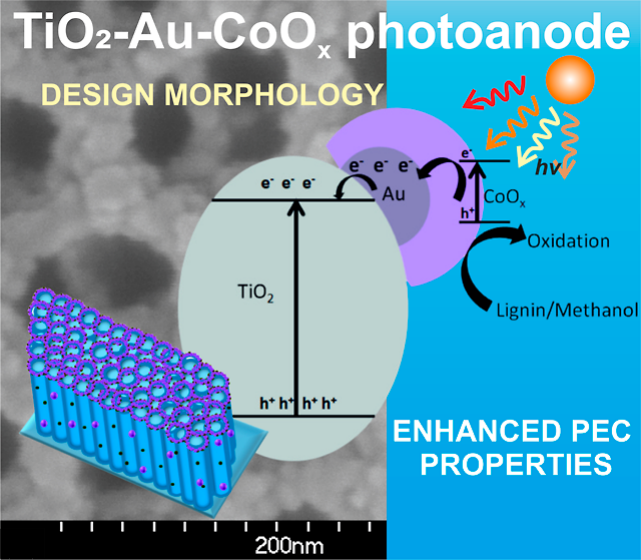

One-dimensional (1D) ordered TiO_2_ nanotubes exhibit exceptional charge transfer capabilities, making them suitable candidates for constructing visible-light-active photoanodes in selective PEC oxidation reactions. Herein, we employed a facile and easily scalable electrochemical method to fabricate Au-CoO_*x*_-deposited ordered TiO_2_ nanotubular array photoanodes. The improved visible light absorption capacity of TiO_2_-Au-CoO_*x*_, with unhampered 1D channels and the controlled integration of Au between TiO_2_ and CoO_*x*_, along with their synergistic interaction, have been identified as the most promising strategy for enhanced PEC performance, as evidenced by an IPCE of 3.7% at 450 nm. Furthermore, the robust interfacial charge transfer pathway from CoO_*x*_ to the TiO_2_ surface via the Au mediator promotes the migration of photogenerated electrons and enables the accumulation of holes on the surface of CoO_*x*_. These holes are then efficiently utilized by oxidants such as methanol or lignin to generate value-added products, highlighting the potential of this system for advanced PEC applications.

## 1 Introduction

Converting solar energy into chemical energy through various techniques, such as photocatalytic (PC) and photoelectrochemical (PEC) processes, represents an environmentally friendly and sustainable approach. It provides a clean and renewable means for energy storage and production, thereby contributing to a greener energy landscape.^1−3^ In recent years, PEC catalysis has been widely employed not only for water-splitting reactions but also for CO_2_ reduction, ammonia synthesis, alcohol oxidation, organic conversion reactions, and more, all achieved at a low potential under mild conditions.^4−9^ In particular, organic transformation reactions like oxidation reactions via PEC have gained more momentum as they could be a greener route than traditional methods for the selective oxidation process. From both an energy generation and value-added products perspective, PEC hydrogen generation or other reduction reactions are now often combined with alcohol oxidation reactions,^1,9−12^ biomass oxidation,^13−16^ and organic pollutant degradation,^17,18^ serving as substitutes for the kinetically unfavorable water oxidation.^8^ Furthermore, this selective oxidation of organics offers the potential to synthesize higher value-added products and simultaneously produces green fuel, further enhancing its sustainability.^19,20^ For example, Cha et al. reported a PEC system capable of generating hydrogen in the cathodic compartment and producing highly valuable acids from biomass in the anodic compartment.^16^ However, using photogenerated charge carriers to drive these highly selective oxidation processes can be challenging. Therefore, to achieve commercial viability, the various organic oxidation reactions needed to be thoroughly investigated regarding production yields and selectivity with a good understanding of reaction mechanisms. For example, Mesa et al. demonstrated methanol oxidation to formaldehyde over a hematite photoanode, achieving nearly unity Faradic efficiency.^8^ Moreover, Zhang et al. reported a selective conversion of biomass-derived benzyl alcohol to benzaldehyde with nearly 99% efficiency and selectivity over a TiO_2_-based photoanode.^21^ In another example, Li et al. achieved an efficient photoelectrocatalytic conversion of lignin model compounds using BiVO_4_-based photoelectrodes.^15^ However, for the smooth operation of these types of organic oxidation reactions, photoelectrocatalysts with favorable sunlight harvesting capabilities, good stability, nontoxicity, higher oxidizing capacity, and natural abundance are essential.

Among various possible heterogeneous catalysts, TiO_2_-based materials have gained widespread use as powerful catalysts for various PEC oxidation processes.^22−26^ Their characteristic properties, such as high abundance, stability over the broad pH range, higher energetic photogenerated holes, and most importantly, higher resistance to photocorrosion, make them one of the most promising photoanode materials.^27,28^ However, their wide optical band gap limits their ability to absorb visible light, and they typically exhibit low charge carrier mobility and high electron–hole recombination rates, which retard their photoefficiency.^29^ The properties of TiO_2_ can be significantly improved when it is controlled at the nanoscale, leading to the fabrication of nanopillars, nanotubes, nanosheets, or rods with controlled porosity, gaining huge scientific interest.^30−37^ 1D materials provide a direct pathway for electron transfer along their length, enhancing the lifetime of photogenerated electrons by allowing swifter migration from the site of generation to the collection point. This, in turn, improves the charge collection efficiency of the photoelectrode. Additionally, 1D materials reduce charge pair recombination by minimizing the encounter of electrons with holes. They also demonstrate enhanced light absorption capability by prolonging the path length of incident light within the structure.^37^ Consequently, there is a growing demand for high-throughput, cost-effective methods to produce such ordered nanoarrays. Regarding this, the synthesis of ordered nanotubular TiO_2_ (NTs) with a high surface area and surface defects via a self-organized anodization of a Ti metal substrate is found to be a model material for different PEC oxidation processes.^2,31^ The 1D structure of NTs can enhance the electronic and ionic conductance by providing a short diffusion pathway for electrons and ions, effectively reducing electron–hole recombination.^31−37^ However, a limitation of nanoporous anodic TiO_2_ is its low visible light absorption ability, necessitating the modulation of its band structure to eliminate this flaw.

One potential strategy, constructing heterostructures by integrating with another visible light-absorbing material, has been explored to improve the PEC performance of TiO_2_ by extending the spectral absorption range and promoting charge separation efficiency.^38,39^ For example, Wu et al. prepared the Au/TiO_2_ photoanode, where the resulting Au/TiO_2_ NTs exhibited enhanced charge separation on TiO_2_ due to the strong interaction between TiO_2_ nanotubes and uniformly dispersed Au nanoparticles.^40^ Tao et al. developed a uniform cluster-sensitized Ni_2_P-TiO_2_ nanotube array heterostructure for a highly efficient PEC urea oxidation reaction.^41^ Lin et al. demonstrated a 4.3-fold increase in PEC performance compared to monomeric TiO_2_ by constructing CeO_2_/TiO_2_ heterojunctions through the chemical deposition of CeO_2_ nanoparticles onto TiO_2_ NTs. This improvement was attributed to optimized energy bands and enhanced visible light absorption.^42^ Hung et al. deposited a thin film of lower band gap Co_3_O_4_ onto TiO_2_ NTs, which exhibited exceptional photoelectrochemical properties compared to neat TiO_2_ NTs. The improved PEC performance can be attributed to the well-regulated Co_3_O_4_ coating layer, which increased visible light absorption and preserved a sizable specific surface area at the electrolyte interface.^43^ Therefore, there is a need for the development of reliable TiO_2_ NTs-based photoanodes with enhanced visible light absorption ability and charge-transfer efficiency for PEC applications. In this study, we synthesized a TiO_2_-Au-CoO_*x*_ nanotube array photoanode for the photoelectrocatalytic oxidation of methanol and lignin. The prepared photoanode exhibited excellent oxidation photocurrent with a lower onset and good stability in PEC measurements. This remarkable photoelectrocatalytic performance can be attributed to the robust electronic interaction between the Au-CoO_*x*_ moiety with TiO_2_ NTs, combined with the unique 1D nanotube array structure, resulting in highly efficient charge generation and separation, as evidenced by different characterization methods and electrochemical analysis. Furthermore, the proper introduction of Au-CoO_*x*_ shell has been identified as another important strategy for enhancing the PEC activity of TiO_2_-Au-CoO_*x*_, particularly when compared to its binary counterpart, i.e., TiO_2_-CoO_*x*_ and neat TiO_2_.

## 2 Experimental Section

### 2.1 Materials Preparation

In brief, a titanium foil (99.5% purity) of 0.25 mm thickness from Alfa Aesar was precut into coupons (1 cm × 2 cm) and degreased in acetone and ethanol. Then, these Ti samples were electrochemically polished for 90 s in a mixed solution of hydrofluoric acid, acetic acid, and sulfuric acid at a constant current density of 70 mA/cm^2^, followed by chemical polishing for 10 s in a mixture of hydrofluoric acid and nitric acid until a mirror finish was exposed. The rear surface and edges of the sample were then insulated with an acid-resistant paint coat. Anodization was carried out in a two-electrode cell, with the titanium foil serving as the working electrode and a larger unpolished Ti foil (5 × 4 cm) serving as the counter electrode. The Ti samples were clamped 2 cm apart from the counter electrode. The anodic oxide layer was produced in three steps at 20 °C in an ethylene glycol solution containing NH_4_F (0.38 wt %) and H_2_O (1.79 wt %). The first and second anodizing steps lasted 3 h, while the duration of the last step was only 10 min for developing amorphous TiO_2_ nanotubular arrays (NTs) on the Ti foil.^31,32,34^ Following the anodizing procedure, the samples were calcined at 400 °C for 1 h to develop a crystalline TiO_2_ anatase structure. Next, before proceeding with Co deposition, some samples undergo Au deposition using the sputtering technique.^44−48^ The sputter coater Quorum Q150T S equipped with a thickness monitor was used for deposition of a 2 nm thick Au layer over anatase TiO_2_ with an estimated sputtering speed of 10 nm/min. Cobalt electrodeposition was carried out using linear sweep voltammetry (LSV) (PalmSens4 potentiostat, PalmSens BV, Houten, The Netherlands) at room temperature (∼20 °C) in a three-electrode setup. TiO_2_/TiO_2_-Au served as the working electrode, while Pt mesh and a saturated calomel electrode (SCE) acted as the counter and reference electrodes, respectively. Cathodic polarization was applied from −0.1 to −1.5 V vs. SCE with a constant scan rate of 50 mV/s. Electrodeposition baths containing 1 M H_3_BO_3_ acid and different concentrations of CoSO_4_·7H_2_O (0.25, 0.5, and 0.75 M) were freshly prepared. Finally, the deposited Co underwent heat treatment at 400 °C for 1 h. The samples were named as TiO_2_-Au-CoO_*x*_ (0.25 M), TiO_2_-Au-CoO_*x*_ (0.5 M), and TiO_2_-Au-CoO_*x*_ (0.75 M) according to the concentration of CoSO_4_ in the electrolyte.

### 2.2 Materials Characterization

The X-ray diffraction patterns of all of the prepared samples were characterized using a Rigaku Miniflex II with a monochromator equipped with Cu (Kα) radiation (λ = 1.5418 Å) from 20° to 60° at a scan rate of 5°/min. The topology and chemical composition of synthesized materials were characterized using a field emission scanning electron microscope (FE-SEM/EDS, Hitachi S-4700 with a Noran System 7, Tokyo, Japan). The TEM and HRTEM images were captured by using a FEI TecnaiOsiris transmission electron microscope operated at an accelerating voltage of 200 kV. UV–visible diffuse reflectance spectra were obtained from a Lambda 750S spectrophotometer (PerkinElmer) equipped with an integrating sphere in the range of 250–800 nm. The AES/XPS spectrometer (Microlab 350, Thermo Electron) was used for monitoring the chemical composition, utilizing the XPS functions of the device with a lateral resolution of 2 ×5 mm^2^. XPS spectra were excited using Alka (*h*ν = 1486.6 eV) radiation as a source. Survey and high resolution spectra were recorded with 100 and 40 eV pass energy, respectively. A smart background subtraction was applied to obtain the XPS signal intensity. The peaks were fitted by using an asymmetric Gaussian/Lorentzian mixed function. The measured binding energies were corrected with reference to the energy of C 1s at 284.7 eV. Data acquisition and processing were carried out using Avantage based data system software (ver. 5.9911, Thermo Fisher Scientific).

### 2.3 Photoelectrochemical Measurements

Photoelectrochemical studies were carried out using a PST electrochemical workstation (PalmSens4, PalmSens BV, Houten, The Netherlands) equipped with a standard three-electrode cell (as described before, annealed materials served as working electrodes) and a 150 W Xe lamp. The shuttered LSV curves were recorded in 0.1 M KNO_3_ (pH 6.6) during anodic polarization from −1.5 to 1 V vs SCE (−0.85–1.65 vs RHE) with a scan rate of 10 mV/s and a step of 0.05 mV. Chronoamperometric (CA) measurements were conducted at a constant potential of 1 V vs SCE. PEC tests using monochromatic light in the range of 300–550 nm (with a 10 nm step) were conducted at 1.65 V vs RHE using a photoelectric spectrometer equipped with the 150 W xenon arc lamp and combined with a potentiostat (Instytut Fotonowy, Krakow, Poland).

The incident photon to current efficiency (IPCE) values were calculated based on the following formula 1(34,38)
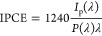
1where 1240 is a constant (W nm/A), *I*_p_ is the photocurrent density (A/m^2^) at the wavelength λ (nm), and *P* is the incident power density of light (W/m^2^) at λ.

The Mott–Schottky analysis was conducted in the same setup, with cathodic polarization applied at 1000 Hz under dark conditions. The flat band potential (*E*_FB_) was calculated by extrapolating the straight region of the Mott–Schottky plot to the potential axis. Then, the donor density (*N*_d_) was estimated based on eq 2(49)
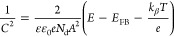
2where ε is the dielectric constant, ε_0_ is the permittivity of the vacuum, *e* is the electron charge, *A* is the active area, *E* is the applied potential, *k*_β_ is the Boltzmann constant, and *T* is the absolute temperature.

### 2.4 Photoelectrochemical Methanol and Lignin Oxidation Reaction Setup

The oxidation process was carried out in a single-compartment Teflon cell with a quartz window, filled with an aqueous electrolyte (0.1 M KNO_3_ + 20 vol % or 20 ppm lignin, for formaldehyde quantification 0.1 M KNO_3_/KOH + 95 vol % methanol), and equipped with three electrodes containing the anodized TiO_2_ heterostructure photoelectrode as the working electrode. All experiments were carried out at room temperature and pressure. Further, a 150 W Xe lamp (Instytut Fotonowy, Krakow, Poland) was used as a light source, and the illumination intensity near the photoelectrode was calculated to be 100–120 mW/cm^2^ at a fixed distance of 10 cm. To observe the PEC oxidation activity over our synthesized materials, we performed CV and LSV analyses over a photovoltage window of (−1.5–1 V vs SCE or −0.85–1.65 V vs. RHE) with a scan rate of 5 mV/s, while CA was performed at a constant voltage of 1 V vs SCE.

### 2.5 Formaldehyde Determination

Formaldehyde, a product of methanol oxidation, was quantified through spectrophotometric measurements using 4-amino-3-hydrazino-5-mercapto-1,2,4-triazole, commonly known as Purpald (≥99% purity, Sigma-Aldrich). Typically, 0.1 g of Purpald and 10 mL of 0.1 M NaOH were thoroughly dissolved in a 25 mL volumetric flask. Subsequently, the stock solution of formaldehyde was added to this solution and left open for 0.5 h with constant stirring at room temperature, resulting in the development of a violet color as formaldehyde formed a complex with the organic reagent in a basic medium. Following this, water was added to adjust the volume, and the solution was analyzed at 549 nm using a Lambda 750S spectrophotometer (PerkinElmer). A calibration curve was constructed for estimation purposes, utilizing a standard formaldehyde solution ranging from 0 to 5 ppm.^8,50^

## 3 Results and Discussion

**Figure 1:**
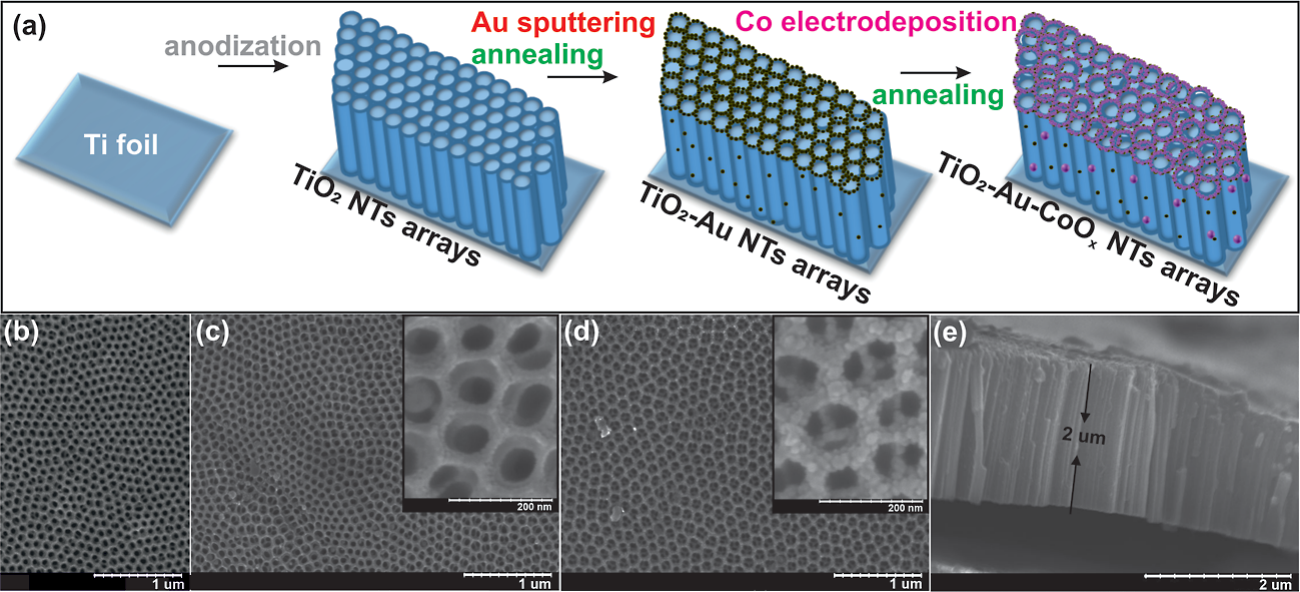
(a) Schematic illustration of the TiO_2_-Au-CoO_*x*_ fabrication process. SEM images of (b) TiO_2_, (c) TiO_2_-CoO_*x*_, (d) TiO_2_-Au-CoO_*x*_ (inset high-resolution SEM images), and (e) cross-sectional SEM image of TiO_2_-Au-CoO_*x*_.

In this work, the TiO_2_-Au-CoO_*x*_ photoanode was prepared by combining electrochemical synthesis techniques and gold dewetting,^35,36,48^ as schematically presented in Figure 1a. Initially, a three-step anodization procedure was followed, and a 1.6 ± 0.2 μm thick array of TiO_2_ nanotubes were formed over Ti foil.^31,34^ Subsequently, cobalt was deposited onto TiO_2_ nanotubes via cathodic electrodeposition using an acidic CoSO_4_ solution at various concentrations to prepare TiO_2_-CoO_*x*_ photoanodes. Meanwhile, for making the TiO_2_-Au-CoO_*x*_ catalyst, before depositing Co, a 2 nm thick Au layer was deposited over bare TiO_2_ nanotube arrays by a sputtering technique. Finally, after calcination, TiO_2_-Au-CoO_*x*_ and TiO_2_-CoO_*x*_ were resulted for further analysis. The morphology, topology, and composition of the synthesized materials were studied using scanning electron microscopy (SEM) and transmission electron microscopy (TEM). The phase composition was investigated by using X-ray photoelectron spectroscopy (XPS) and X-ray diffraction (XRD) to assess the purity of the catalyst. Optical and semiconducting properties were investigated by UV–Vis diffuse reflection spectroscopy (UV–vis DRS) and Mott–Schottky analysis, respectively. Finally, the photoelectrochemical properties of the obtained materials were tested in the sunlight-induced oxidation of water, methanol, and lignin.

As depicted in Figure 1b, the SEM image shows that the TiO_2_ nanotube arrays are formed with a distinct honeycomb-like morphology, and the cross-sectional image reveals a close-packed, vertically aligned, and relatively smooth nanotube surface with a thickness of 1.6 ± 0.2 μm (Figure S1).^33−35^ The results for TiO_2_-CoO_*x*_ and TiO_2_-Au-CoO_*x*_ (Figure 1c,d, respectively) show that the small particles deposited over the top of TiO_2_ without filling the pores of the arrays. After the deposition of Au and CoO_*x*_ particles, the thickness is calculated to be 2.1 ± 0.1 μm, while very few particles are seen to be inside the nanotubes, as evidenced by the cross-sectional SEM image in Figure 1e. This observation confirms that a majority of Au/CoO_*x*_ particles are deposited solely on the top surface of nanotubes. Since the nanotubes are well preserved and there is no formation of big particles or clogging of the nanochannels, hence, the transfer of charges across the interface between the inner wall of the tube and the electrolyte will definitely be promoted.^43^ Furthermore, both Au/CoO_*x*_ and Co fully cover the top surface of the TiO_2_ NTs. The EDS elemental mapping depicted in Figure S2 confirms the even distribution of Au and Co over the TiO_2_ surface, demonstrating the successful preparation of the TiO_2_-Au-CoO_*x*_ nanotubular array structure.

**Figure 2:**
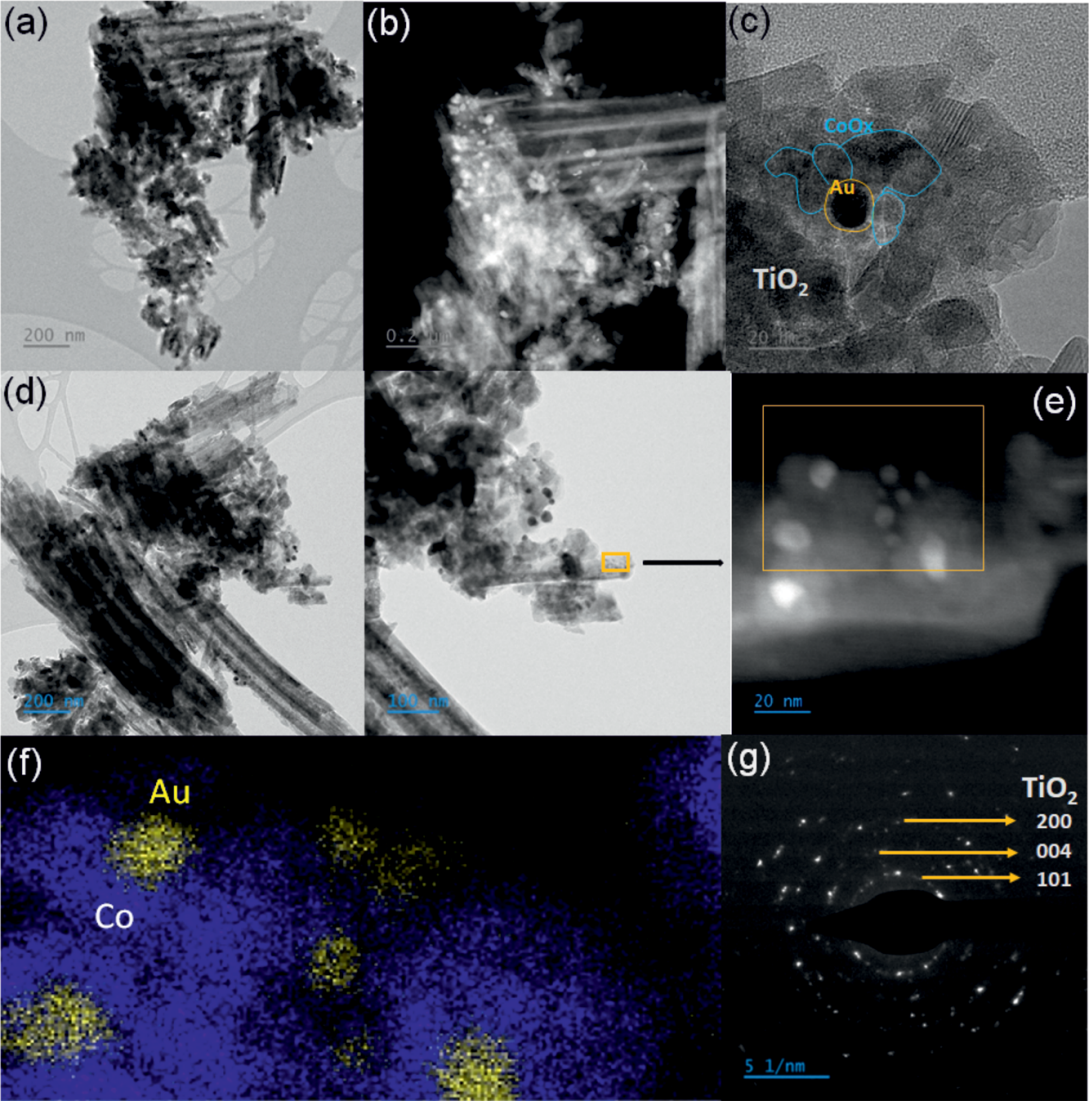
(a,b) Bright field and dark field TEM of TiO_2_-Au-CoO_*x*_, (c) HRTEM images showing lattice fringes of Au, Co_3_O_4_, and TiO_2_ in TiO_2_-Au-CoO_*x*_, (d) low-resolution and high-resolution TEM images of TiO_2_-Au-CoO_*x*_, (e,f) HAADF-STEM image of selected area and its corresponding EDS scan, and (g) SAED diffraction pattern of TiO_2_-Au-CoO_*x*_.

Further internal microstructures and the arrangement of Au and CoO_*x*_ over TiO_2_ are visualized using high-angle annular dark-field scanning transmission electron microscopy (HAADF-STEM). Figure S3 shows a representative HAADF-STEM image of anodized TiO_2_ NTs, revealing a hollow tubular-like structure with a smooth tube wall. Notably, the tube wall consists of two layers, consistent with previous observations.^51^ The TEM image of TiO_2_-Au-CoO_*x*_ is presented in Figure 2, where the materials are observed as agglomerated nanotubular structures obtained by scraping from the Ti foil. As can be seen from Figure 2a, most of the nanotubes are broken and do not have any material over them. However, in some areas, smaller particles are visible as bright spots at the ends of TiO_2_ tubes, as depicted in Figure 2b, indicating the potential presence of Au and CoO_*x*_. Furthermore, in the HRTEM image (Figure 2c), lattice fringes corresponding to different crystal planes are observed. A lattice fringe spacing of 0.35 nm, corresponding to the (101) plane of TiO_2_, is detected. Additionally, two distinct fringes are observed: one with a spacing of 0.235 nm, corresponding to the (111) plane of Au,^26^ and another with a spacing of 0.24 nm, associated with irregular particles covering the Au and corresponding to electrodeposited cobalt species, such as the (311) plane of Co_3_O_4_^52−54^ (Figure S4a). To gain deeper insights into the elemental distribution and chemical environment of Au@CoO_*x*_, electron energy loss spectroscopy (EELS) was conducted under the STEM mode. HAADF-STEM images of the selected area and the corresponding EDS line scan analysis along the highlighted line direction are shown in Figures 2d–f and S4b–e. The EDS elemental profile of the selected area, indicated by the yellow square in Figure 2f, confirms the presence of Co (marked in blue) and Au (marked in yellow). It can be observed that most of the Au nanoparticles are surrounded by Co, providing evidence for the coverage of CoO_*x*_ nanoparticles around Au.^55^ A STEM-EDS mapping of the samples (Figure S4b,c) in that region reveals the presence of Ti, Au, oxygen, and cobalt, further affirming the successful preparation of TiO_2_-Au-CoO_*x*_. Additionally, the EELS line scan profile (Figure S4d) for two Au particles demonstrates that Au is predominantly covered with a CoO_*x*_ shell.

Evaluation of the Co-L_3,2_ edge fine structure, as shown in Figure S4e, reveals two peaks for Co-L_3_ (∼778 eV) and Co-L_2_ (∼793.5 eV), corresponding to transitions from 2p_3/2_ and 2p_1/2_ to unoccupied 3d states of CoO_*x*_, respectively. These peaks exhibit a difference of 15.5 eV, with the average Co L_3_/L_2_ peak intensity ratio around 2.5 corresponding to Co/Co_3_O_4_, suggesting that cobalt is present in the form of CoO_*x*_.^52,54^ Furthermore, the selected area diffraction pattern (SAED) in Figure 2g displays clear concentric rings corresponding to the diffraction pattern of neat TiO_2_, consistent with the XRD analysis. However, intermediary bright dots observed between the (004) and (200) TiO_2_ planes, forming rings, result from diffraction originating from the Au and cobalt oxide samples.

**Figure 3:**
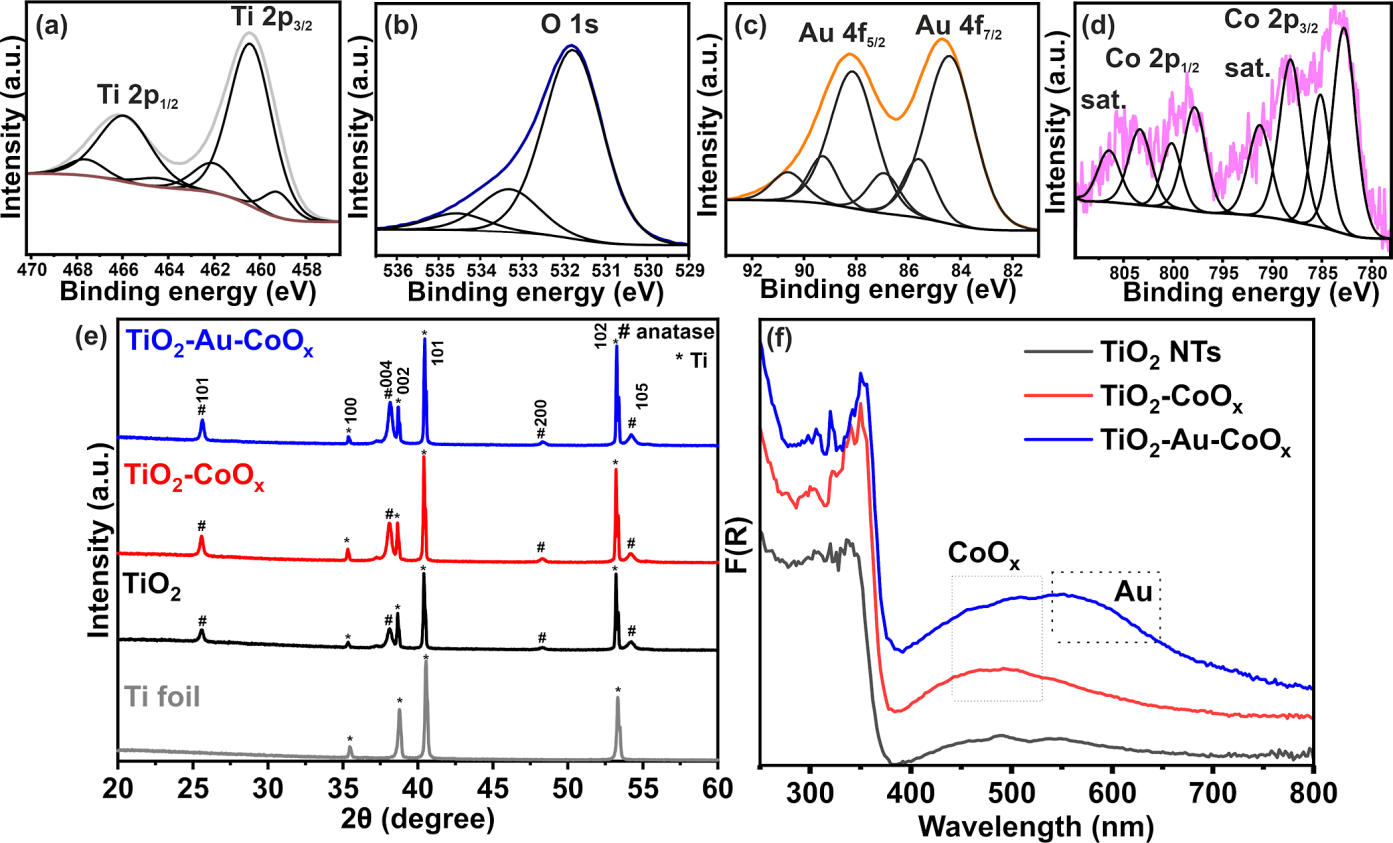
High-resolution XPS spectra of (a) Ti 2p, (b) O 1s, (c) Au 4f, and (d) Co 2p. XRD patterns (e) of Ti foil, TiO_2_ NTs, TiO_2_ NTs-CoO_*x*_, and TiO_2_ NTs-Au-CoO_*x*_. (f) Kubekla-Munk function of TiO_2_ NTs, TiO_2_ NTs-CoO_*x*_, and TiO_2_ NTs-Au-CoO_*x*_.

The surface composition of TiO_2_-Au-CoO_*x*_ was analyzed by XPS (the survey scan is presented in Figure S5). High-resolution spectra confirm the presence of titanium (Figure 3a), oxygen (Figure 3b), gold (Figure 3c), and cobalt (Figure 3d). The analysis of these spectra reveals the presence of titanium oxide, with the Ti 2p_3/2_ spin–orbital at 458.8 eV and oxygen O 1s at 530.2 eV. This signal can be assigned to metal oxides, as low concentrations of Au_2_O_3_ and CoO can also be identified.^56^ Gold appears in its metallic form, as evidenced by the peaks centered at 84.0 and 87.7 eV. The main peak originating from cobalt at the binding energy of 781.2 eV can be assigned to Co^2+^ as CoO or Co(OH)_2_.^56^ The estimated contents of gold and cobalt on the material’s surface were 3.5 and 0.1 at. %, respectively. Moreover, the XPS spectra of C 1s (Figure S5, inset) and O 1s indicate the presence of carbon and also carbon-oxygen functional groups, which correspond to typical surface contaminants.^57^

The crystal structure was characterized by XRD, as illustrated in Figure 3e. As expected, reflections from titanium (JCPDS card no. 05–0682) with crystal planes of (100), (002), (101), (102), and anatase phase (JCPDS card no. 21–1272) (101), (004), (200), and (105) planes were detected.^32^ In addition, the crystal plane (004) emerged as the main peak for nanotube arrays and showed the strongest diffraction peak. The results reveal no indication of impurities. No additional diffraction peaks either for CoO_*x*_ or Au phases are observed in TiO_2_-CoO_*x*_ and TiO_2_-Au-CoO_*x*_, confirming the uniform distribution of small amounts of Au and CoO_*x*_ over the top of TiO_2_. Furthermore, no noticeable shift in the anatase phase is observed upon deposition, but a significant change in the relative intensity and area of the XRD peak, especially at 38.1°, is evident, as depicted in Figures 3e and S6. Both Au and Co_3_O_4_ exhibit major planes (111)^26^ and (311),^52−54^ respectively, around this diffraction angle, i.e., 38.1°, Consequently, a substantial increase in peak area and intensity is observed in the XRD diffraction pattern, specifically along the (004) plane of TiO_2_, which confirms the successful deposition of Au-CoO_*x*_ onto TiO_2_ and formation of a stable anode material.^58^

The optical properties, especially the ability to absorb visible light, of the prepared materials were analyzed by using UV–Vis diffuse reflectance spectroscopy (Figure 3f). As expected, TiO_2_ NTs exhibit an absorption edge around 380 nm, primarily absorbing in the UV region. However, a broad hump is also observed in the visible region, attributed to scattering effects caused by pores or cracks in the nanotube arrays, as reported in the literature.^43,59^ Both TiO_2_-CoO_*x*_ and TiO_2_-Au-CoO_*x*_ demonstrate enhanced light absorption abilities, indicating improved light-capturing ability under both UV and visible regions due to the effective interaction between CoO_*x*_ and Au-CoO_*x*_ with the TiO_2_ interface, confirming the successful formation of an improved photoanode. As seen in Figure 3f, an absorption band designated for CoO_*x*_ is evident for both TiO_2_-CoO_*x*_ and TiO_2_-Au-CoO_*x*_ samples, but a localized surface plasmon resonance (LSPR) band at about 550–650 nm can only be seen in the TiO_2_-Au-CoO_*x*_ sample.^26^ Furthermore, a broad LSPR hump is seen for the TiO_2_-Au-CoO_*x*_ (0.25 M) sample at around 500–650 nm. When the electrolyte concentration of CoO_*x*_ is increased up to 0.75 M, the additional absorption at around 400–550 nm increases gradually, which is due to the deposition of CoO_*x*_ particles^43^ (Figure S7a). According to the Tauc’s plot, as shown in Figure S7b, neat TiO_2_ shows a single band gap of 3.36 eV, while there is a slight change in the band gap of the composite. This change might be due to some minute doping of Co ions in the crystal lattice of TiO_2_, but the alteration is very negligible.

**Figure 4:**
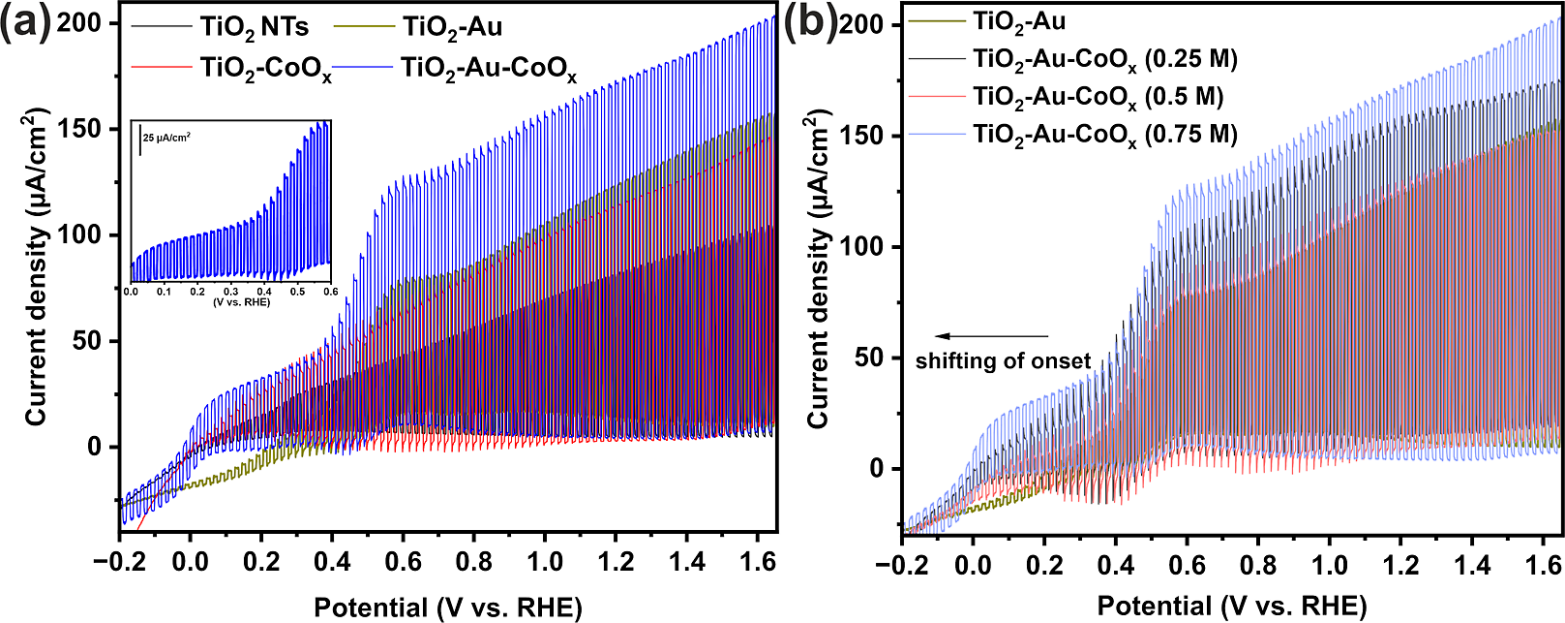
(a) Shuttered LSV curves of TiO_2_ NTs, TiO_2_-Au, TiO_2_-CoO_*x*_, and TiO_2_-Au-CoO_*x*_ and (b) shuttered LSV plots of TiO_2_-Au and TiO_2_-Au-CoO_*x*_ of different concentrations.

To evaluate the effect of deposited species on the PEC properties of TiO_2_, we measured the photocurrent of TiO_2_-Au, TiO_2_-CoO_*x*_, and TiO_2_-Au-CoO_*x*_ nanotube arrays, comparing them with neat TiO_2_ NTs. The PEC measurements were conducted in a 0.1 M KNO_3_ electrolyte under simulated sunlight with an AM 1.5G filter. The linear sweep voltammetry (LSV) curves of TiO_2_ and modified TiO_2_ nanotube arrays, in a potential range of – 1.5 to 1 V vs SCE (from −0.85 to 1.65 V vs RHE) with chopped light, are shown in Figure 4a. As can be seen, the TiO_2_ NT photoanode exhibits a very low dark current, while under illumination, it shows a pronounced photocurrent starting nearly around 0.05 V and continues to increase up to 104 μA/cm^2^ at 1.65 V vs RHE with an increase in the applied voltage. A remarkable increase in the photocurrent density was observed for all of the modified samples, surpassing the photocurrent density of neat TiO_2_. The photocurrent density is as high as 203 μA/cm^2^ for TiO_2_-Au-CoO_*x*_ and 146 μA/cm^2^ for TiO_2_-CoO_*x*_ at 1.65 V vs RHE. The observed improvement mainly stems from the efficient charge transfer induced by the external electric field and the effective charge separation across the interface. Additionally, in some cases, there is a response in photocurrent before the onset potential. This phenomenon may be attributed to the presence of trapped photogenerated charges caused by light exposure.^42,60^

All the materials exhibit a slight negative onset potential compared to neat TiO_2_, indicating that surface modification with CoO_*x*_ facilitates band bending at the electrode–electrolyte interface and promotes a faster charge transfer process.^61^ Both TiO_2_-CoO_*x*_ and TiO_2_-Au-CoO_*x*_ show nearly identical onset potentials, while TiO_2_-Au demonstrates a small anodic shift in it. To verify the reason, we further examined the LSV curves for TiO_2_-Au-CoO_*x*_ obtained at various CoO_*x*_ concentrations and compared them with TiO_2_-Au as shown in Figure 4b. It was observed that with an increase in CoO_*x*_ concentration, there is a gradual cathodic shift of the onset potential. This shift can be attributed to the gradual formation of a CoO_*x*_ shell around the Au particles, which insulates Au from the electrolyte. This effect has been described by Li et al. in their study on the TiO_2_-Au-CdS material.^62^ The observation of a small anodic shift in the onset potential for TiO_2_-Au, despite displaying substantially higher photocurrent than TiO_2_, seems to contradict the typical behavior expected when an Au-containing electrode is immersed in the electrolyte, which typically results in a negative shift of the Fermi level due to charge equilibration.^62^ This positive shift may be connected to the existence of a pseudocurrent plateau between the potential range of 0.4 to 0.65 V vs RHE (Figure 4). Notably, this characteristic oxidation plateau is detected for Au-decorated samples only, as no such peak is observed for neat TiO_2_ or TiO_2_-CoO_*x*_ in that potential window. This could be related to the photoelectrochemical generation of holes on noble nanoparticles.^63^ When the LSV scans of neat and Au-modified TiO_2_ are compared, it is evident that the former exhibits an almost perfect square shape for the successive on–off cycles, while the latter shows cathodic and anodic spikes. Cathodic photocurrent spikes typically occur when accumulated holes in the space charge layer recombine with bulk electrons during irradiation, while positive photocurrent spikes are associated with the accumulation of holes in the electrode space charge layer during irradiation.^64,65^ The cathodic spikes, observed in light off conditions, were seen in the range of 0 to 0.6 V vs. RHE for TiO_2_-Au, TiO_2_-CoO_*x*_, and TiO_2_-Au-CoO_*x*_ samples (Figure S8a,b). Importantly, the positive current spikes are larger than negative ones, indicating that a considerable percentage of the photogenerated charge carriers undergo recombination through the surface states, leading to a reduction in photocurrent.^63^ These pseudoplateaus and spikes are not present in TiO_2_ due to a negligible population of trap states. In the TiO_2_-CoO_*x*_ LSV profile, the pseudoplateau is absolutely absent, but high cathodic and anodic spikes are present. For TiO_2_-Au-CoO_*x*_, both pseudoplateau and spikes are present, indicating that surface states play a dominant role in the PEC properties of Au-modified TiO_2_. Furthermore, note that the intensity of these spikes changes depending on the electrode polarization. After 0.6 V vs RHE, negative spikes diminish completely, while positive spikes steadily decrease with further polarization in the anodic direction (Figure 4a, inset). Then, above about 1 V vs. RHE, an almost perfectly squared shape is seen, suggesting that the TiO_2_-Au-CoO_*x*_ sample has fewer holes accumulated at the electrode–electrolyte interface, and the recombination rate of photogenerated charge carriers is minimal.^64,65^ Among the different CoO_*x*_ concentration-loaded TiO_2_-Au-CoO_*x*_ samples, 0.75 M shows lower anodic spikes and negligible cathodic spikes (Figure S8c), further confirming that effective loading of cobalt species over Au is crucial for improved PEC performance.

**Figure 5:**
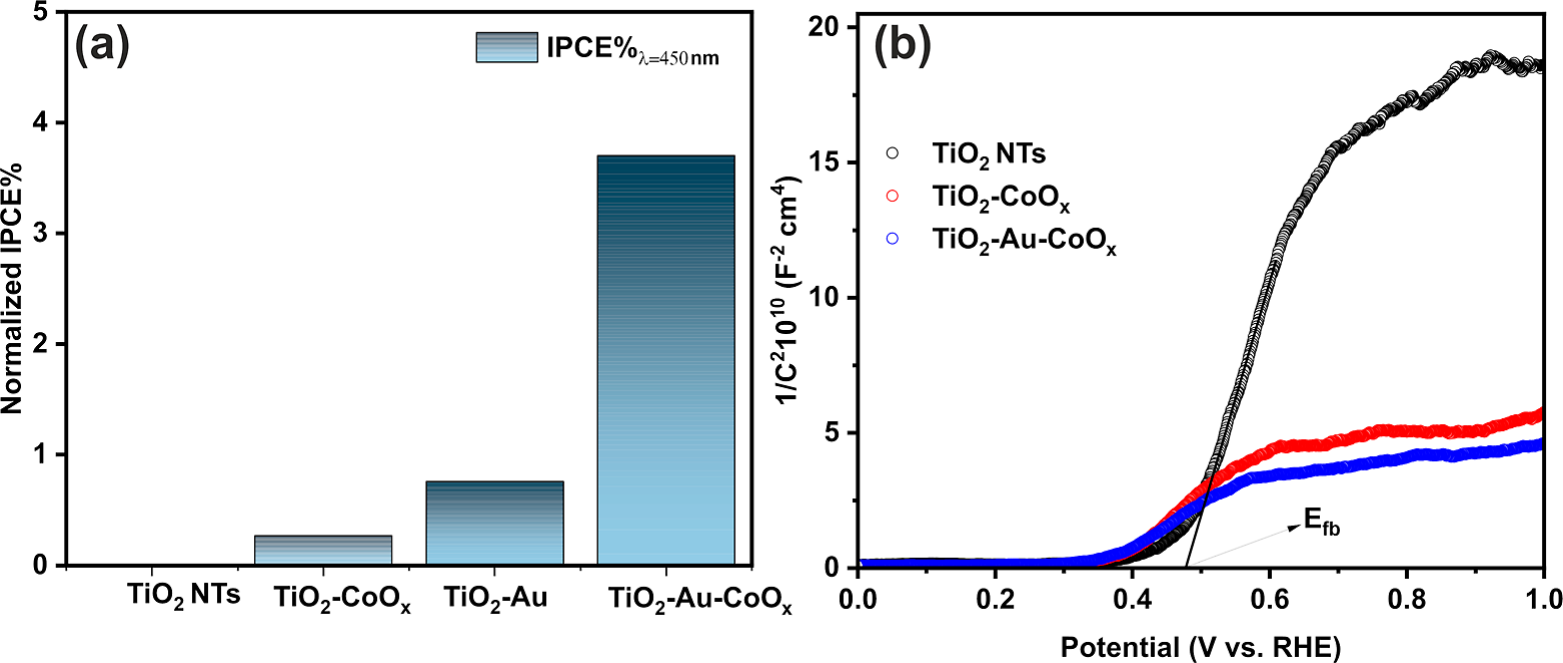
(a) IPCE % at 450 nm and (b) Mott–Schottky plot of TiO_2_ NTs, TiO_2_-CoO_*x*_, and TiO_2_-Au-CoO_*x*_.

The incident photon to current efficiency (IPCE) values for the photoanodes were calculated and are shown in Figure S9. For the visible light region, i.e., 450 nm (Figure 5a), the TiO_2_-Au-CoO_*x*_ photoanode exhibits the highest IPCE % of 3.7%, surpassing both TiO_2_-CoO_*x*_ as well as neat TiO_2_. Moreover, in the UV-region of 360–420 nm, our synthesized material also excels in efficiency because of the synergistic interaction of the three components.

To further understand the PEC performance and analyze the excellent performance of the TiO_2_-Au-CoO_*x*_ photocatalyst, electrochemical impedance spectroscopy measurements were conducted at a frequency of 1 kHz in the dark. The Mott–Schottky measurements of pristine and modified samples are presented in Figure 5b. All samples exhibit a positive slope, as expected for n-type semiconductors, in the corresponding Mott–Schottky plots. The calculated carrier densities follow the order TiO_2_-Au-CoO_*x*_ > TiO_2_-CoO_*x*_ > TiO_2_. Both Co and Au-CoO_*x*_ samples lead to a significant enhancement of donor density compared to that of TiO_2_, which might be another factor contributing to their improved PEC activity. As the concentration of donor rises, more electrons populate the conduction band, causing the Fermi level to approach the edge of the conduction band. This shift accelerates charge separation at the electrode–electrolyte interface by amplifying the energy gradient, thus enhancing the bending of energy bands. Moreover, the modified samples exhibit a slight negative change in flat band potential with respect to TiO_2_, similar to the photocurrent onset potential, which shows a slightly more cathodic shift for the modified samples in the LSV curves.^66,67^ It generally arises from the difference in the surface catalytic properties of the electrode in the presence of light. In the present work, the flat band potential in the Mott–Schottky plot remained the same for both TiO_2_-CoO_*x*_ and TiO_2_-Au-CoO_*x*_, as evidenced by the same onset in the LSV profile. The lack of Fermi level equilibrating was not surprising, as the gold nanoparticles were fully covered with CoO_*x*_ nanoparticles, isolating them from the liquid electrolyte.^62^ Hence, Au incorporation between TiO_2_ and CoO_*x*_ has been proven to be an effective strategy for improving the PEC activity.

**Figure 6:**
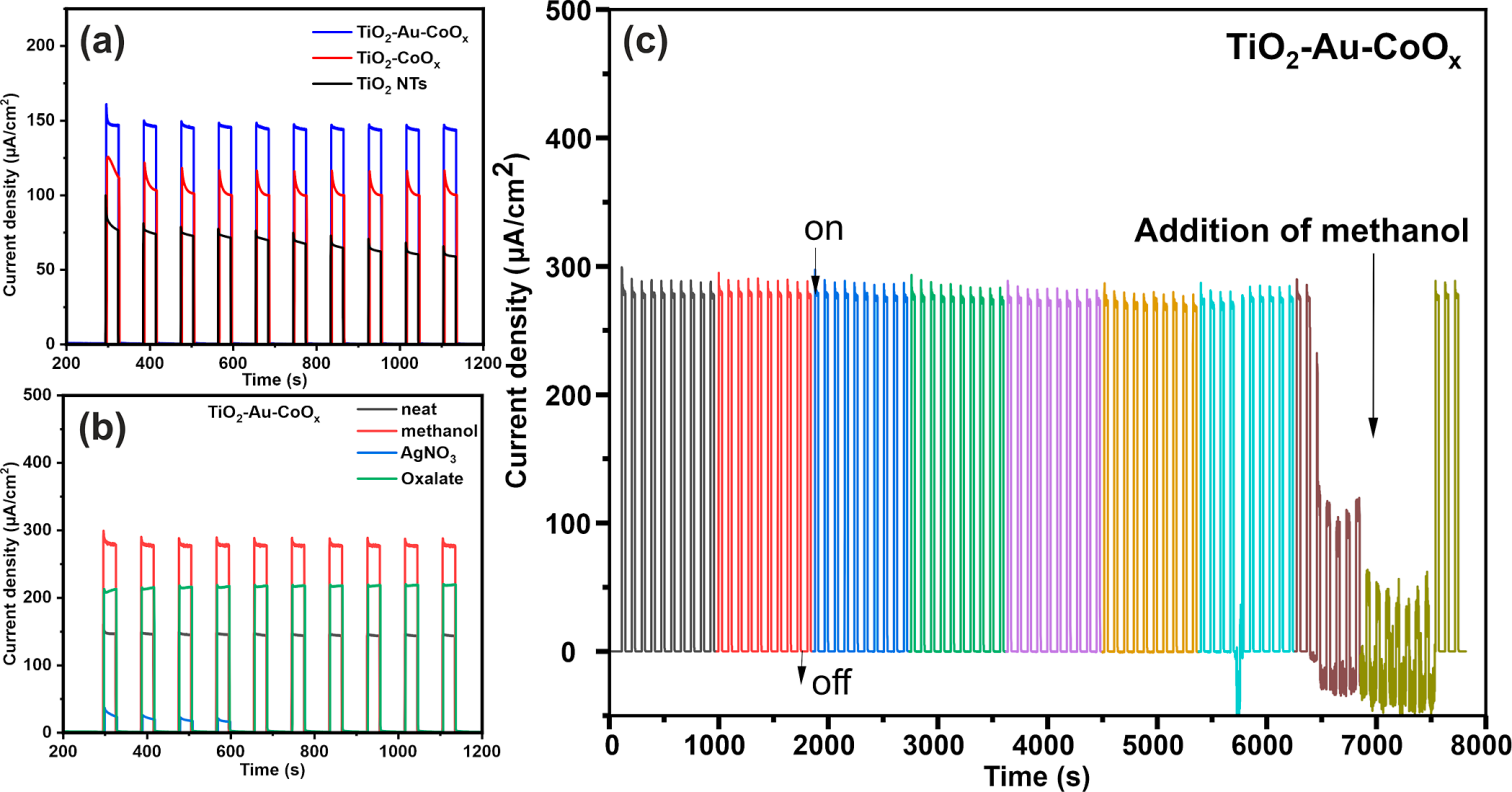
On–off CA plots (a) recorded for TiO_2_ NTs, TiO_2_-CoO_*x*_, and TiO_2_-Au-CoO_*x*_. On–off CA plots (b) recorded for the TiO_2_-Au-CoO_*x*_ photoanode in the presence of different scavengers. (c) PEC response of TiO_2_-Au-CoO_*x*_ materials in the presence of methanol as a scavenger (8000 s window).

To further identify the improved photogenerated charge pair transfer and separation performance, chronoamperometry (CA) profiles were measured with a bias potential of +1 V vs. SCE (Figure 6). As shown in figures, with the switchable light on and off, the photocurrent signal displays a reversible behavior, suggesting good stability and reproducibility of all materials in light conditions. Furthermore, all the materials exhibit higher photocurrent density compared to neat TiO_2._ The increased photoresponse confirms that the built-in and external electric fields work together to speed up the separation of charges in modified TiO_2_. From both LSV and CA measurements, TiO_2_-Au-CoO_*x*_ exhibited a higher photocurrent compared to neat TiO_2_ and is thus concluded to be the best material.

**Figure 7:**
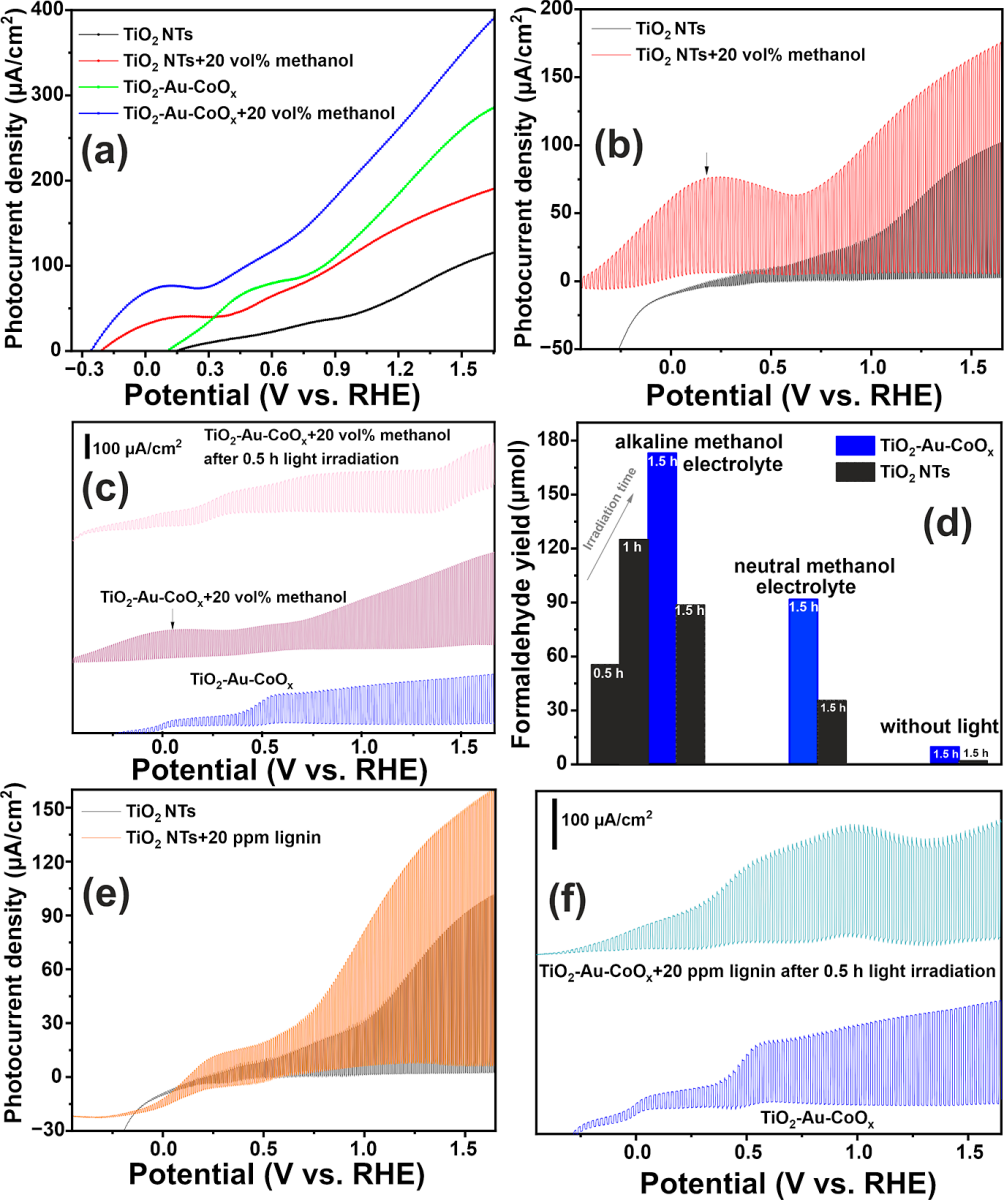
(a) LSV curves of TiO_2_ and TiO_2_-Au-CoO_*x*_ in light illumination, shuttered LSV plots of (b) TiO_2_ and (c) TiO_2_-Au-CoO_*x*_ in the absence and presence of methanol, (d) formaldehyde production yield in different conditions, and (e,f) shuttered LSV curves for TiO_2_ and TiO_2_-Au-CoO_*x*_ in the presence and absence of lignin.

Both TiO_2_ and TiO_2_-Au-CoO_*x*_ show long-term stability during the 1200 s window. However, neat TiO_2_ experiences around a 10% loss in initial photocurrent due to the accumulation of holes at the surface in operation, resulting in the photocorrosion of TiO_2_. In contrast, the TiO_2_-Au-CoO_*x*_ photoanode shows a very stable photoresponse, with a loss of only 2%. The higher stability and low loss may be attributed to the fact that Au and Au-CoO_*x*_ nanoparticles completely cover the top of the nanotubes, effectively separating electrons and holes and thus protecting the surface from photocorrosion. Further, we tested the efficiency of our TiO_2_-Au-CoO_*x*_ material in an electrolyte containing different scavengers^68^ (Figure 6b). For hole scavengers (methanol and sodium oxalate), the photoanode exhibits a higher photocurrent density, reducing electron-hole pair recombination. However, for the electron scavenger, the photocurrent drastically diminishes after 600 s, as silver nanoparticles completely cover the surface of the photoanode, thereby reducing light penetration. Among methanol and sodium oxalate (hole scavengers), our synthesized material exhibited a higher photocurrent in methanol. Hence, we further studied the effect of methanol on the stability of the material, as shown in Figure 6c. In 20 vol % methanol, the material exhibits a very high stability up to 6000 s of chopped light irradiation with a loss of only 3%. However, interestingly, after that, there is a sudden decrease in the photocurrent density up to 90%. To restore the activity, we added methanol again to the electrolyte, and the activity was restored. To investigate the cause of this alteration, normal and shuttered LSV and CA responses of TiO_2_ and TiO_2_-Au-CoO_*x*_ photoanodes in 0.1 M KNO_3_ + 20 vol % methanol under light illumination were recorded, as shown in Figures 7 and S10. The use of hole scavengers, such as methanol, has proven effective in reducing electron/hole recombination losses, acting as an alternative to the application of anodic potentials. In some studies, a photocurrent doubling mechanism is observed as a result of methanol scavenging, generating two long-lived conduction band electrons per scavenged hole, thereby causing a doubling in the photocurrent. Systematical studies have shown that in aliquant methanol, oxidation leads to the formation of formaldehyde and ultimately to CO_2_. Studies on oxidation of methanol in neutral medium at semiconductors such as CeO_2_, WO_3_, In_2_O_3_, BiVO_4_, C_3_N_4_, etc., have explored the adsorption of methanol on the catalyst surface, followed by the formation of a CH_3_O^•^ radical and its subsequent oxidation with a valence band holes.^8,69−73^ Similarly, in our case, we observed a significant increase in photocurrent density for TiO_2_, accompanied by the appearance of an oxidation hump in the potential window of −0.3 to 0.3 V. The evident shift of the onset potential to the cathodic direction in the presence of methanol indicates a higher hole transfer rate, possibly due to the change in the injection barrier (kinetic or thermodynamic) for the holes to the electrolyte.^68^ This observation aligns well with findings reported in the literature,^8,68−73^ reflecting changes in the injection barrier that alter the observed photocurrent onset potential of the material. For TiO_2_-Au-CoO_*x*_, a similar observation was reported, with a much lower onset potential and higher photocurrent, nearly doubling and approaching 300 μA/cm^2^. These results are consistent with the existing literature.^8,68−71^ Furthermore, the rectangular shape of the shuttered LSV plot without spikes in Figure 7a,b recorded under illumination indicates that holes are effectively quenched by oxidant species, accelerating effective photoinduced charge separation and transfer. The figure signifies that, according to the results, a nearly doubled photocurrent, an oxidation hump, and a lower onset potential indicate improved kinetics for methanol oxidation compared to the OER.^72^ Similarly, in our study, a photocurrent doubling mechanism occurred, strengthening the conclusion that our synthesized material is highly active for neutral medium alcohol oxidation. However, after continuous 0.5 h light irradiation, these features disappear, supporting our CA analysis, which indicates that methanol is completely exhausted in the system (Figure 7c).

Further, to analyze the products formed during methanol PEC oxidation, we employed colorimetric analysis. After 0.5 h of irradiating light, we attempted to measure the formaldehyde concentration. Both TiO_2_ and TiO_2_-Au-CoO_*x*_ photoanodes showed a low amount of formaldehyde. To quantitatively analyze the formaldehyde formation, we conducted additional experiments using 95 vol % methanol in 0.1 M KNO_3_ and 0.1 M KOH at 1 V vs. SCE under light illumination, as described by Mesa et al.^8^ Interestingly, in both media, TiO_2_-Au-CoO_*x*_ exhibited a substantially higher formaldehyde yield compared to neat TiO_2_, suggesting its enhanced charge separation and transfer ability. In the alkaline medium, with an increase in light irradiation time, the yield of formaldehyde gradually increased, and after 1.5 h of light irradiation, the nanohybrid photoanode exhibited a formaldehyde yield of 173 μmol/L, while in the neutral medium, it showed a yield of 91.8 μmol/L, as shown in Figure 7d. Furthermore, the photoanodes demonstrated exceptional performance in a neutral medium as well. However, the higher efficiency in an alkaline medium might be attributed to the higher adsorption of methanol on the catalyst surface and the production of more free oxide radicals participating in methanol oxidation.^74^ Additionally, we tested methanol oxidation without illuminating light at 1 V vs. SCE and found a very low production efficiency, confirming that light is essential for carrying out the methanol oxidation reaction. A comparison of PEC methanol activity of different reported photoanodes is presented in Table S1.

Lignin, a polyphenolic biopolymer and a key component of lignocellulosic biomass, holds significant potential for conversion into valuable aromatic chemicals. In recent years, lignin oxidation to useful chemicals in PEC half-cells has gathered considerable attention.^10,15^ To investigate the capability of our synthesized photoelectrode for lignin oxidation, we conducted LSV and CA analyses in a three-electrode configuration under simulated solar light. From the LSV profile in Figure 7e,f, we observed an increase in the photocurrent upon the addition of lignin for both TiO_2_ and TiO_2_-Au-CoO_*x*_ photoelectrodes. The results suggest that lignin provides electrons to the photoelectron-activated materials, possibly acting as an electron donor. Furthermore, the lignin oxidation process appears to be more favorable than the water oxidation reaction. For the TiO_2_-Au-CoO_*x*_ photoelectrodes, with an increment in photocurrent, a plateau before 1.15 V vs. RHE appears when lignin is present in the electrolyte. This anodic hump occurs prior to the oxygen evolution (1.23 V), and this oxidation process might be governed by a chemisorbed active oxygen mechanism, as previously reported in the literature.^75^

**Figure 8:**
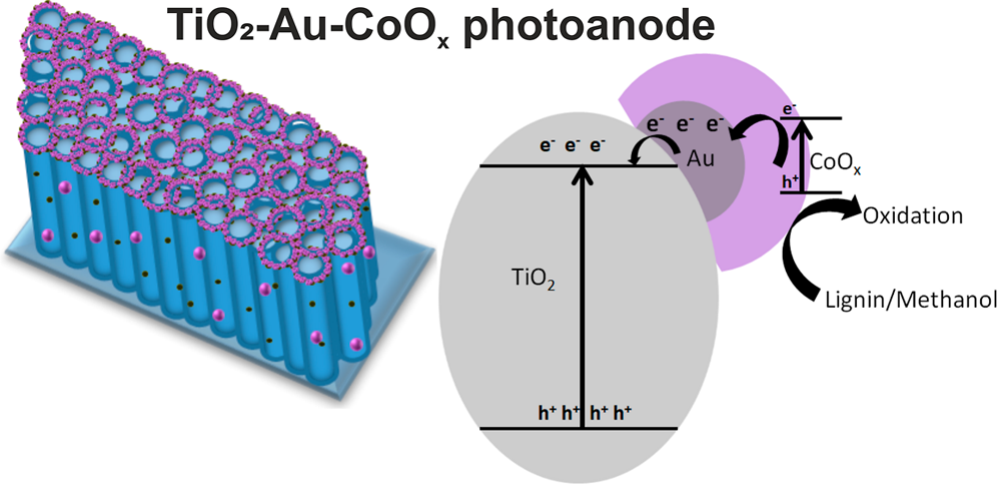
Schematic illustration of the charge transfer mechanism in TiO_2_-Au-CoO_*x*_.

In brief, the TiO_2_-Au-CoO_x_ photoelectrode demonstrates improved PEC performance and proves to be more active for both methanol and lignin oxidation compared to neat TiO_2_. Based on all the observations mentioned above, the charge separation and transfer mechanism are illustrated in Figure 8. The controlled construction of TiO_2_-Au-CoO_*x*_ and the interface between TiO_2_-Au and Au-CoO_*x*_ play crucial roles in the efficient charge separation and transfer processes, thereby showing improved PEC performance. In brief, when TiO_2_, Au, and CoO_*x*_ came in close proximity, their individual Fermi level and band edges are rearranged, as evidenced by the negative shifting of onset as well as the flat band as seen from LSV and Mott–Schottky plots. This negative change of the band potential is more pronounced in TiO_2_-Au-CoO_*x*_ than pristine TiO_2_, confirming the upward band bending that creates an interface for swifter charge migration. Upon UV–Vis light irradiation, both TiO_2_ and CoO_*x*_ are excited and produce electrons in the conduction band minimum (CBM) and holes in the valence band maximum. The produced electrons of CoO_*x*_ swiftly migrate to the CBM of TiO_2_ and then to the external circuit via Ti-Au-Co interfaces, and the migration process suffers less resistance due to 1D nanotubular channels and Au mediators. Meanwhile, the energetic holes at the surface of TiO_2_ move toward the CoO_*x*_ surface, where they participate in the methanol and lignin oxidation processes. We also assume that the holes accumulated at the TiO_2_ surface become more accessible to the CoO_*x*_ surface with increased biasing. Hence, combined holes are readily used in the oxidation process more efficiently, as seen from doubled photocurrent and higher methanol and lignin oxidation percentages for TiO_2_-Au-CoO_*x*_ than pristine TiO_2_. The generation and separation efficiency of photogenerated charge pairs are significantly improved because of the synergistic effect of TiO_2_, Au, and CoO_*x*_, as evidenced from different PEC analyses. The proper control of generation of the Au-CoO_*x*_ shell and its interfaces helps in reducing charge transfer resistance and increasing the accelerated charge transfer process. Next, we assume that Au not only acts as an electron mediator but also participates in the PEC oxidation activity by generating plasmonic hot electrons and holes, as evidenced from enhanced IPCE at the 450–550 nm range window, but the later effect is very low. As reported in many studies, when Au comes into immediate contact with TiO_2_, plasmonic hot electrons are generated and can transfer from the plasmonic Au metal to the conduction band of semiconductor TiO_2_ via Schottky contact at a higher wavelength of light irradiation.^60,76,77^ Hence, under lower wavelength light irradiation, the electron transfer process is accelerated by Au mediator, while under higher wavelength irradiation, a small amount of the produced hot holes participates in PEC oxidation activity.

## 4 Conclusions

In this study, TiO_2_-Au-CoO_*x*_ and TiO_2_-CoO_*x*_ photoanodes were obtained using the electrochemical anodization technique and Au sputtering, followed by an electrochemical deposition process. The PEC performance of the photoanodes was investigated, and TiO_2_-Au-CoOx exhibited a higher photocurrent density and showed good activity for methanol and lignin oxidation. The superior PEC performance of TiO_2_-Au-CoO_*x*_, with an IPCE of 3.7% at 450 nm, is attributed to improved visible light absorption ability. The incorporation of Au between TiO_2_ and CoO_*x*_ has been proven to be an effective strategy for accelerating charge separation and the transfer process. Furthermore, the proper formation of the Au@CoO_*x*_ shell structure, with controlled CoO_*x*_ deposition and an unhampered nanotubular structure, was found to be essential for an effective pathway for the swift migration of electrons from CoO_*x*_ to TiO_2_ and then to the circuit. The accumulated electrons at the surface of CoO_*x*_ were utilized in the oxidation process. Hence, the use of nanotubular, high surface area TiO_2_, and its efficient combination with Au-CoO_*x*_ could be employed in future exploration for possible applications in different mediums, lignin as well as the methanol PEC oxidation reaction. This approach has the potential to evolve as the best strategy to replace the unfavorable water oxidation reaction in green fuel generation.

## Supporting Information Available

Cross-sectional SEM image of TiO_2_ NTs, EDS distribution images of TiO_2_-Au-CoO_*x*_, low and high resolution HAADF-STEM images of TiO_2_ NTs, STEM-EDS color mapping of TiO_2_-Au-CoO_*x*_, EDS-EELS line scan analysis, Co L edge EELS, XPS survey scan, XRD patterns of different CoO_*x*_ concentrations TiO_2_ NTs-Au-CoO_*x*_, Kubelka–Munk plot for TiO_2_-Au-CoO_*x*_ of different concentrations, Band gap plots, Cathodic and anodic spikes of photoanodes, IPCE spectra of tested materials, and On–off CA plot of TiO_2_ and TiO_2_-Au-CoO_*x*_ in KNO_3_ and KNO_3_ containing methanol and lignin (PDF)

## Abbreviations

1D: one-dimensional
NTs: ordered nanotubular arrays
IPCE: incident to photon conversion efficiency
SEM: scanning electron microscopy
TEM: transmission electron microscopy
XRD: X-ray diffraction
DRS-UV–Vis: UV–Vis diffuse reflectance spectroscopy
EDS: energy dispersive spectra
HAADF-STEM: high angle annular dark field-scanning transmission electron microscopy
EELS: electron energy loss spectroscopy
SAED: selected area diffraction pattern
LSPR: localized surface plasmon resonance
LSV: linear sweep voltammetry
CA: chronoamperometry
SCE: standard calomel electrode
RHE: reversible hydrogen electrode

## References

[ref1] Niu F.; Zhang P.; Zhang Z.; Zhou Q.; Li P.; Liu R.; Li W.; Hu K. Ultrathin corrugated nanowire TiO_2_ as a versatile photoanode platform for boosting photoelectrochemical alcohol and water oxidation. J. Mater. Chem. A 2023, 11 (8), 4170–4182. 10.1039/D2TA09613G.

[ref2] Hou H.; Shao G.; Wang Y.; Wong W. Y.; Yang W. Insights on advanced substrates for controllable fabrication of photoanodes toward efficient and stable photoelectrochemical water splitting. Carbon Energy 2024, 6 (4), e373 10.1002/cey2.373.

[ref3] Wu H.; Tan H. L.; Toe C. Y.; Scott J.; Wang L.; Amal R.; Ng Y. H. Photocatalytic and photoelectrochemical systems: similarities and differences. Adv. Mater. 2020, 32 (18), 1904717 10.1002/adma.201904717. 31814196

[ref4] Song K.; Hou H.; Gong C.; Gao F.; Zhang D.; Zhi F.; Yang W.; He F. Enhanced solar water splitting of BiVO_4_ photoanodes by in situ surface band edge modulation. J. Mater. Chem. A 2022, 10 (42), 22561–22570. 10.1039/D2TA06141D.

[ref5] Han G. H.; Bang J.; Park G.; Choe S.; Jang Y. J.; Jang H. W.; Kim S. Y.; Ahn S. H. Recent advances in electrochemical, photochemical, and photoelectrochemical reduction of CO_2_ to C^2+^ products. Small 2023, 19 (16), 2205765 10.1002/smll.202205765. 36592422

[ref6] Sultana S.; Paramanik L.; Mansingh S.; Parida K. Robust photoelectrochemical route for the ambient fixation of dinitrogen into ammonia over a nanojunction assembled from ceria and an iron boride/phosphide cocatalyst. Inorg. Chem. 2022, 61 (1), 131–140. 10.1021/acs.inorgchem.1c02504. 34936349

[ref7] Huang S.; Feng F.; Huang R. T.; Ouyang T.; Liu J.; Liu Z. Q. Activating C–H bonds by tuning Fe sites and an interfacial effect for enhanced methanol oxidation. Adv. Mater. 2022, 34 (50), 2208438 10.1002/adma.202208438. 36216372

[ref8] Mesa C. A.; Kafizas A.; Francàs L.; Pendlebury S. R.; Pastor E.; Ma Y.; Le Formal F.; Mayer M. T.; Grätzel M.; Durrant J. R. Kinetics of photoelectrochemical oxidation of methanol on hematite photoanodes. J. Am. Chem. Soc. 2017, 139 (33), 11537–11543. 10.1021/jacs.7b05184. 28735533 PMC5594441

[ref9] Karjule N.; Phatake R. S.; Barzilai S.; Mondal B.; Azoulay A.; Shames A. I.; Volokh M.; Albero J.; García H.; Shalom M. Photoelectrochemical alcohols oxidation over polymeric carbon nitride photoanodes with simultaneous H_2_ production. J. Mater. Chem. A 2022, 10 (31), 16585–16594. 10.1039/D2TA03660F. PMC9365238 36091884

[ref10] Li S.; Park S.; Sherman B. D.; Yoo C. G.; Leem G. Photoelectrochemical approaches for the conversion of lignin at room temperature. Chem. Commun. 2023, 59 (4), 401–413. 10.1039/D2CC05491D. 36519448

[ref11] Miao Y.; Li Z.; Song Y.; Fan K.; Guo J.; Li R.; Shao M. Surface active oxygen engineering of photoanodes to boost photoelectrochemical water and alcohol oxidation coupled with hydrogen production. Appl. Catal., B 2023, 323, 122147 10.1016/j.apcatb.2022.122147.

[ref12] Antón-García D.; Edwardes Moore E.; Bajada M. A.; Eisenschmidt A.; Oliveira A. R.; Pereira I. A.; Warnan J.; Reisner E. Photoelectrochemical hybrid cell for unbiased CO_2_ reduction coupled to alcohol oxidation. Nat. Synth. 2022, 1 (1), 77–86. 10.1038/s44160-021-00003-2.

[ref13] Lu X.; Xie S.; Yang H.; Tong Y.; Ji H. Photoelectrochemical hydrogen production from biomass derivatives and water. Chem. Soc. Rev. 2014, 43 (22), 7581–7593. 10.1039/C3CS60392J. 24599050

[ref14] Zhang Y.; Zhao G.; Zhang Y.; Huang X. Highly efficient visible-light-driven photoelectro-catalytic selective aerobic oxidation of biomass alcohols to aldehydes. Green Chem. 2014, 16 (8), 3860–3869. 10.1039/C4GC00454J.

[ref15] Li T.; Mo J. Y.; Weekes D. M.; Dettelbach K. E.; Jansonius R. P.; Sammis G. M.; Berlinguette C. P. Photoelectrochemical decomposition of lignin model compound on a BiVO_4_ photoanode. ChemSusChem 2020, 13 (14), 3622–3626. 10.1002/cssc.202001134. 32369260

[ref16] Cha H. G.; Choi K. S. Combined biomass valorization and hydrogen production in a photoelectrochemical cell. Nat. Chem. 2015, 7 (4), 328–333. 10.1038/nchem.2194. 25803471

[ref17] Koo M. S.; Cho K.; Yoon J.; Choi W. Photoelectrochemical degradation of organic compounds coupled with molecular hydrogen generation using electrochromic TiO_2_ nanotube arrays. Environ. Sci. Technol. 2017, 51 (11), 6590–6598. 10.1021/acs.est.7b00774. 28445067

[ref18] Liu S. S.; Xing Q. J.; Chen Y.; Zhu M.; Jiang X. H.; Wu S. H.; Dai W.; Zou J. P. Photoelectrochemical degradation of organic pollutants using BiOBr anode coupled with simultaneous CO_2_ reduction to liquid fuels via CuO cathode. ACS Sustain. Chem. Eng. 2019, 7 (1), 1250–1259. 10.1021/acssuschemeng.8b04917.

[ref19] Lhermitte C. R.; Sivula K. Alternative oxidation reactions for solar-driven fuel production. ACS Catal. 2019, 9 (3), 2007–2017. 10.1021/acscatal.8b04565.

[ref20] Qi M. Y.; Conte M.; Anpo M.; Tang Z. R.; Xu Y. J. Cooperative coupling of oxidative organic synthesis and hydrogen production over semiconductor-based photocatalysts. Chem. Rev. 2021, 121 (21), 13051–13085. 10.1021/acs.chemrev.1c00197. 34378934

[ref21] Zhang R.; Shao M.; Li Z.; Ning F.; Wei M.; Evans D. G.; Duan X. Photoelectrochemical catalysis toward selective anaerobic oxidation of alcohols. Chem.—Eur. J. 2017, 23 (34), 8142–8147. 10.1002/chem.201701107. 28485855

[ref22] Pu Y. C.; Ling Y.; Chang K. D.; Liu C. M.; Zhang J. Z.; Hsu Y. J.; Li Y. Surface passivation of TiO_2_ nanowires using a facile precursor-treatment approach for photoelectrochemical water oxidation. J. Phys. Chem. C 2014, 118 (27), 15086–15094. 10.1021/jp5041019.

[ref23] Cheng B. Y.; Yang J. S.; Cho H. W.; Wu J. J. Fabrication of an efficient BiVO_4_–TiO_2_ heterojunction photoanode for photoelectrochemical water oxidation. ACS Appl. Mater. Interfaces 2016, 8 (31), 20032–20039. 10.1021/acsami.6b05489. 27454929

[ref24] Chen H.; Chen K. F.; Lai S. W.; Dang Z.; Peng Y. P. Photoelectrochemical oxidation of azo dye and generation of hydrogen via CN co-doped TiO_2_ nanotube arrays. Sep. Purif. Technol. 2015, 146, 143–153. 10.1016/j.seppur.2015.03.026.

[ref25] Li W.; He D.; Hu G.; Li X.; Banerjee G.; Li J.; Lee S. H.; Dong Q.; Gao T.; Brudvig G. W.; Waegele M. M.; et al. Selective CO production by photoelectrochemical methane oxidation on TiO_2_. ACS Cent. Sci. 2018, 4 (5), 631–637. 10.1021/acscentsci.8b00130. 29806010 PMC5968511

[ref26] Xing Y.; Sheng X.; Zhou H.; Wang D.; Chen X.; Feng X. Long and well-separated TiO_2_ nanowire arrays decorated with Au nanoparticles for visible-light-driven photoelectrochemical water splitting. J. Phys. Chem. C 2022, 126 (4), 1966–1971. 10.1021/acs.jpcc.1c10081.

[ref27] Zhou W.; Fu H. Mesoporous TiO_2_: preparation, doping, and as a composite for photocatalysis. ChemCatChem 2013, 5 (4), 885–894. 10.1002/cctc.201200519.

[ref28] Zhang W.; He H.; Li H.; Duan L.; Zu L.; Zhai Y.; Li W.; Wang L.; Fu H.; Zhao D. Visible-light responsive TiO_2_-based materials for efficient solar energy utilization. Adv. Energy Mater. 2021, 11 (15), 2003303 10.1002/aenm.202003303.

[ref29] Daghrir R.; Drogui P.; Robert D. Modified TiO_2_ for environmental photocatalytic applications: a review. Ind. Eng. Chem. Res. 2013, 52 (10), 3581–3599. 10.1021/ie303468t.

[ref30] Song K.; Hou H.; Zhang D.; He F.; Yang W. In-situ cation-exchange strategy for engineering single-atomic Co on TiO_2_ photoanode toward efficient and durable solar water splitting. Appl. Catal., B 2023, 330, 122630 10.1016/j.apcatb.2023.122630.

[ref31] Sulka G. D.; Kapusta-Kołodziej J.; Brzózka A.; Jaskuła M. Fabrication of nanoporous TiO_2_ by electrochemical anodization. Electrochim. Acta 2010, 55 (14), 4359–4367. 10.1016/j.electacta.2009.12.053.

[ref32] Kapusta-Kołodziej J.; Syrek K.; Pawlik A.; Jarosz M.; Tynkevych O.; Sulka G. D. Effects of anodizing potential and temperature on the growth of anodic TiO_2_ and its photoelectrochemical properties. Appl. Surf. Sci. 2017, 396, 1119–1129. 10.1016/j.apsusc.2016.11.097.

[ref33] Üzer E.; Kumar P.; Kisslinger R.; Kar P.; Thakur U. K.; Zeng S.; Shankar K.; Nilges T. Vapor deposition of semiconducting phosphorus allotropes into TiO_2_ nanotube arrays for photoelectrocatalytic water splitting. ACS Appl. Nano Mater. 2019, 2 (6), 3358–3367. 10.1021/acsanm.9b00221.

[ref34] Sulka G. D.; Kapusta-Kołodziej J.; Brzózka A.; Jaskuła M. Anodic growth of TiO_2_ nanopore arrays at various temperatures. Electrochim. Acta 2013, 104, 526–535. 10.1016/j.electacta.2012.12.121.

[ref35] Yoo J.; Lee K.; Schmuki P. Dewetted Au films form a highly active photocatalytic system on TiO_2_ nanotube-stumps. Electrochem. Commun. 2013, 34, 351–355. 10.1016/j.elecom.2013.07.008.

[ref36] Licklederer M.; Mohammadi R.; Nguyen N. T.; Park H.; Hejazi S.; Halik M.; Vogel N.; Altomare M.; Schmuki P. Dewetted Au nanoparticles on TiO_2_ surfaces: evidence of a size-independent plasmonic photoelectrochemical response. J. Phys. Chem. C 2019, 123 (27), 16934–16942. 10.1021/acs.jpcc.9b02769.

[ref37] Yu Z.; Liu H.; Zhu M.; Li Y.; Li W. Interfacial charge transport in 1D TiO_2_ based photoelectrodes for photoelectrochemical water splitting. Small 2021, 17 (9), 1903378 10.1002/smll.201903378. 31657147

[ref38] Sołtys-Mróz M.; Syrek K.; Wiercigroch E.; Małek K.; Rokosz K.; Raaen S.; Sulka G. D. Enhanced visible light photoelectrochemical water splitting using nanotubular FeOx-TiO_2_ annealed at different temperatures. J. Power Sources 2021, 507, 230274 10.1016/j.jpowsour.2021.230274.

[ref39] Vahidzadeh E.; Zeng S.; Manuel A. P.; Riddell S.; Kumar P.; Alam K. M.; Shankar K. Asymmetric multipole plasmon-mediated catalysis shifts the product selectivity of CO_2_ photoreduction toward C2+ products. ACS Appl. Mater. Interfaces 2021, 13 (6), 7248–7258. 10.1021/acsami.0c21067. 33539093

[ref40] Wu L.; Li F.; Xu Y.; Zhang J. W.; Zhang D.; Li G.; Li H. Plasmon-induced photoelectrocatalytic activity of Au nanoparticles enhanced TiO_2_ nanotube arrays electrodes for environmental remediation. Appl. Catal., B 2015, 164, 217–224. 10.1016/j.apcatb.2014.09.029.

[ref41] Tao Y.; Ma Z.; Wang W.; Zhang C.; Fu L.; Zhu Q.; Li Y.; Li G.; Zhang D. Nickel phosphide clusters sensitized TiO_2_ nanotube arrays as highly efficient photoanode for photoelectrocatalytic urea oxidation. Adv. Funct. Mater. 2023, 33 (9), 2211169 10.1002/adfm.202211169.

[ref42] Lin S. W.; Tong M. H.; Chen Y. X.; Chen R.; Zhao H. P.; Jiang X.; Yang K.; Lu C. Z. CeO_2_/TiO_2_ heterojunction nanotube arrays for highly efficient visible-light photoelectrochemical water splitting. ACS Appl. Energy Mater. 2023, 6 (2), 1093–1102. 10.1021/acsaem.2c03723.

[ref43] Huang B.; Yang W.; Wen Y.; Shan B.; Chen R. Co_3_O_4_-modified TiO_2_ nanotube arrays via atomic layer deposition for improved visible-light photoelectrochemical performance. ACS Appl. Mater. Interfaces 2015, 7 (1), 422–431. 10.1021/am506392y. 25493324

[ref44] Grochowska K.; Nedyalkov N.; Karczewski J.; Haryński Ł.; Śliwiński G.; Siuzdak K. Anodic titania nanotubes decorated with gold nanoparticles produced by laser-induced dewetting of thin metallic films. Sci. Rep. 2020, 10, 20506 10.1038/s41598-020-77710-x. 33239673 PMC7688952

[ref45] Nguyen N. T.; Altomare M.; Yoo J.; Schmuki P. Efficient photocatalytic H_2_ evolution: controlled dewetting–dealloying to fabricate site-selective high-activity nanoporous Au particles on highly ordered TiO_2_ nanotube arrays. Adv. Mater. 2015, 27, 3208–3215. 10.1002/adma.201500742. 25872758

[ref46] Nguyen N. T.; Hwang I.; Kondo T.; Yanagishita T.; Masuda H.; Schmuki P. Optimizing TiO_2_ nanotube morphology for enhanced photocatalytic H_2_ evolution using single-walled and highly ordered TiO_2_ nanotubes decorated with dewetted Au nanoparticles. Electrochem. Commun. 2017, 79, 46–50. 10.1016/j.elecom.2017.04.016.

[ref47] Zhou D.; Liu Y.; Zhang W.; Liang W.; Yang F. Au-TiO_2_ nanofilms for enhanced photocatalytic activity. Thin Solid Films 2017, 636, 490–498. 10.1016/j.tsf.2017.06.051.

[ref48] Zhang W.; Liu Y.; Zhou D.; Wen J.; Zheng L.; Liang W.; Yang F. Diffusion kinetics of gold in TiO_2_ nanotube arrays for formation of Au@TiO_2_ nanotube arrays. RSC Adv. 2016, 6, 48580–48588. 10.1039/C6RA08801E.

[ref49] Bak C. H.; Kim K.; Jung K.; Kim J. B.; Jang J. H. Efficient photoelectrochemical water splitting of nanostructured hematite on a three-dimensional nanoporous metal electrode. J. Mater. Chem. A 2014, 2 (41), 17249–17252. 10.1039/C4TA03578J.

[ref50] Jacobsen N. W.; Dickinson R. G. Spectrometric assay of aldehydes as 6-mercapto-3-substituted-s-trizolo (4, 3-b)-tetrazines. Anal. Chem. 1974, 46 (2), 298–299. 10.1021/ac60338a039.

[ref51] Wang D.; Liu L.; Zhang F.; Tao K.; Pippel E.; Domen K. Spontaneous phase and morphology transformations of anodized titania nanotubes induced by water at room temperature. Nano Lett. 2011, 11 (9), 3649–3655. 10.1021/nl2015262. 21786788

[ref52] Ramakrishnan V.; Kim H.; Park J.; Yang B. Cobalt oxide nanoparticles on TiO_2_ nanorod/FTO as a photoanode with enhanced visible light sensitization. RSC Adv. 2016, 6 (12), 9789–9795. 10.1039/C5RA23200G.

[ref53] Li X.; Liu Y.; Sun Q.; Huang W. H.; Wang Z.; Chueh C. C.; Chen C. L.; Zhu Z. Surface engineered CoP/Co_3_O_4_ heterojunction for high-performance bi-functional water splitting electro-catalysis. Nanoscale 2021, 13 (47), 20281–20288. 10.1039/D1NR06044A. 34817488

[ref54] Zhao Y.; Feltes T. E.; Regalbuto J. R.; Meyer R. J.; Klie R. F. In situ electron energy loss spectroscopy study of metallic Co and Co oxides. J. Appl. Phys. 2010, 108 (6), 063704 10.1063/1.3482013.

[ref55] Zhang J.; Zhang Q.; Wang L.; Li X. A.; Huang W. Interface induce growth of intermediate layer for bandgap engineering insights into photoelectrochemical water splitting. Sci. Rep. 2016, 6 (1), 27241 10.1038/srep27241. 27250648 PMC4890116

[ref56] Moulder J. F.; Stickle W. F.; Sobol P. E.; Bomben K. D.; Chastain J. Handbook of X-Ray Photoelectron Spectroscopy: A Reference Book of Standard Spectra for Identification and Interpretation of XPS Data, Physical Electronics Division; Perkin-Elmer Corporation, 1992; pp 82–83.

[ref57] Syrek K.; Gurgul M.; Pisarek M.; Chrabaszcz K.; Malek K.; Sulka G. Synthesis and the visible light activity of anodic CoO_x_-TiO_2_ nanocomposites. J. Phys. Chem. C 2024, 128, 7679–7689. 10.1021/acs.jpcc.4c00142.

[ref58] Manjunatha M.; Reddy G. S.; Mallikarjunaiah K. J.; Damle R.; Ramesh K. P. Determination of phase composition of cobalt nanoparticles using ^59^Co internal field nuclear magnetic resonance. J. Supercond. Nov. Magnetism 2019, 32, 3201–3209. 10.1007/s10948-019-5083-7.

[ref59] Dai G.; Yu J.; Liu G. Synthesis and enhanced visible-light photoelectrocatalytic activity of p– n junction BiOI/TiO_2_ nanotube arrays. J. Phys. Chem. C 2011, 115 (15), 7339–7346. 10.1021/jp200788n.

[ref60] Wu M.; Chen W. J.; Shen Y. H.; Huang F. Z.; Li C. H.; Li S. K. In situ growth of matchlike ZnO/Au plasmonic heterostructure for enhanced photoelectrochemical water splitting. ACS Appl. Mater. Interfaces 2014, 6 (17), 15052–15060. 10.1021/am503044f. 25144940

[ref61] Ma Y.; Hu Y. H. Efficient Ni (OH)_2_/WO_3_ photoanode for photoelectrocatalytic water splitting at low bias. J. Phys. Chem. C 2020, 124 (36), 19447–19456. 10.1021/acs.jpcc.0c04900.

[ref62] Li J.; Cushing S. K.; Zheng P.; Senty T.; Meng F.; Bristow A. D.; Manivannan A.; Wu N. Solar hydrogen generation by a CdS-Au-TiO_2_ sandwich nanorod array enhanced with Au nanoparticle as electron relay and plasmonic photosensitizer. J. Am. Chem. Soc. 2014, 136 (23), 8438–8449. 10.1021/ja503508g. 24836347

[ref63] Gomes Silva C.; Juárez R.; Marino T.; Molinari R.; García H. Influence of excitation wavelength (UV or visible light) on the photocatalytic activity of titania containing gold nanoparticles for the generation of hydrogen or oxygen from water. J. Am. Chem. Soc. 2011, 133 (3), 595–602. 10.1021/ja1086358. 21142160

[ref64] Lipińska W.; Grochowska K.; Ryl J.; Karczewski J.; Siuzdak K. Influence of annealing atmospheres on photoelectrochemical activity of TiO_2_ nanotubes modified with AuCu nanoparticles. ACS Appl. Mater. Interfaces 2021, 13 (44), 52967–52977. 10.1021/acsami.1c16271. 34704439

[ref65] Khan R.; Naveen M. H.; Abbas M. A.; Lee J.; Kim H.; Bang J. H. Photoelectrochemistry of Au nanocluster-sensitized TiO_2_: intricacy arising from the light-induced transformation of nanoclusters into nanoparticles. ACS Energy Lett. 2021, 6 (1), 24–32. 10.1021/acsenergylett.0c02306.

[ref66] Loukopoulos S.; Sakellis E.; Kostakis M. G.; Gerokonstantis D. T.; Tsipas P.; Gardelis S.; Kontos A. G.; Katsaros F. K.; Sideratou Z.; Romanos G. E.; Dimoulas A.; et al. Co-assembled MoS_2_–TiO_2_ inverse opal photocatalysts for visible light-activated pharmaceutical photodegradation. ACS Omega 2023, 8 (37), 33639–33650. 10.1021/acsomega.3c03881. 37744818 PMC10515384

[ref67] Iandolo B.; Zhang H.; Wickman B.; Zorić I.; Conibeer G.; Hellman A. Correlating flat band and onset potentials for solar water splitting on model hematite photoanodes. RSC Adv. 2015, 5 (75), 61021–61030. 10.1039/C5RA10215D.

[ref68] Denisov N.; Yoo J.; Schmuki P. Effect of different hole scavengers on the photoelectrochemical properties and photocatalytic hydrogen evolution performance of pristine and Pt-decorated TiO_2_ nanotubes. Electrochim. Acta 2019, 319, 61–71. 10.1016/j.electacta.2019.06.173.

[ref69] Li S.; Chen F.; Ma T.; Huang H. Vacancy engineered BiVO_4_ photoanode realizes efficient photoelectrochemical CH_3_OH oxidation in near-neutral media: Active site regulation improves HCHO selectivity. Chem. Eng. J. 2023, 467, 143421 10.1016/j.cej.2023.143421.

[ref70] Lu X.; Zheng D.; Zhang P.; Liang C.; Liu P.; Tong Y. Facile synthesis of free-standing CeO_2_ nanorods for photoelectrochemical applications. Chem. Commun. 2010, 46 (41), 7721–7723. 10.1039/c0cc01854f. 20852788

[ref71] Gan J.; Lu X.; Zhai T.; Zhao Y.; Xie S.; Mao Y.; Zhang Y.; Yang Y.; Tong Y. Vertically aligned In_2_O_3_ nanorods on FTO substrates for photoelectrochemical applications. J. Mater. Chem. 2011, 21 (38), 14685–14692. 10.1039/c1jm11774b.

[ref72] Cristino V.; Caramori S.; Argazzi R.; Meda L.; Marra G. L.; Bignozzi C. A. Efficient photoelectrochemical water splitting by anodically grown WO_3_ electrodes. Langmuir 2011, 27 (11), 7276–7284. 10.1021/la200595x. 21542603

[ref73] Li L.; Xiao S.; Li R.; Cao Y.; Chen Y.; Li Z.; Li G.; Li H. Nanotube array-like WO_3_ photoanode with dual-layer oxygen-evolution cocatalysts for photoelectrocatalytic overall water splitting. ACS Appl. Energy Mater. 2018, 1 (12), 6871–6880. 10.1021/acsaem.8b01215.

[ref74] Jiang D.; Zhao H.; Jia Z.; Cao J.; John R. Photoelectrochemical behaviour of methanol oxidation at nanoporous TiO_2_ film electrodes. J. Photochem.Photobio. A: Chem. 2001, 144 (2–3), 197–204. 10.1016/S1010-6030(01)00527-5.

[ref75] Bateni F.; Ghahremani R.; Staser J. A. Electrochemical oxidative valorization of lignin by the nanostructured PbO_2_/MWNTs electrocatalyst in a low-energy depolymerization process. J. Appl. Electrochem. 2021, 51, 65–78. 10.1007/s10800-020-01451-y.

[ref76] Brennan L. J.; Purcell-Milton F.; Salmeron A. S.; Zhang H.; Govorov A. O.; Fedorov A. V.; Gun’ko Y. K. Hot plasmonic electrons for generation of enhanced photocurrent in gold-TiO_2_ nanocomposites. Nanoscale Res. Lett. 2015, 10, 38 10.1186/s11671-014-0710-5. 25852335 PMC4385105

[ref77] Zeng S.; Vahidzadeh E.; VanEssen C. G.; Kar P.; Kisslinger R.; Goswami A.; Zhang Y.; Mahdi N.; Riddell S.; Kobryn A. E.; Gusarov S.; et al. Optical control of selectivity of high rate CO_2_ photoreduction via interband-or hot electron Z-scheme reaction pathways in Au-TiO_2_ plasmonic photonic crystal photocatalyst. Appl. Catal., B 2020, 267, 118644 10.1016/j.apcatb.2020.118644.

