# Supplementary material for: Designing TiO2 Nanotubular Arrays with Au-CoOx Core–Shell Nanoparticles for Enhanced Photoelectrochemical Methanol and Lignin Oxidation
Source: ACS Appl Mater Interfaces. 2024 Sep 4;16(37):49262–74. doi: 10.1021/acsami.4c07498 (PMC11420873; doi:10.1021/acsami.4c07498)
Supplement: Supplementary file 1 — am4c07498_si_001.pdf [file am4c07498_si_001.pdf]

Supporting Information

Designing TiO_2 nanotubular arrays with Au-CoO_x core-shell nanoparticles for enhanced photoelectrochemical methanol and lignin oxidation

Sabiha Sultana[1], Izabela Darowska[1], Marcin Pisarek[2], Grzegorz D. Sulka[1], Karolina Syrek[1]*

[1]Jagiellonian University, Department of Physical Chemistry and Electrochemistry

Gronostajowa 2, 30 - 387 Krakow, Poland

[2]Laboratory of Surface Analysis, Institute of Physical Chemistry, Polish Academy of Sciences,

Kasprzaka 44/52, 01-224 Warsaw, Poland

Keywords: nanotube array, photoelectrochemical oxidation, methanol, lignin,

* Corresponding author. E-mail: syrek@chemia.uj.edu.pl; karolina.syrek@uj.edu.pl

Jagiellonian University, Faculty of Chemistry, Department of Physical Chemistry & Electrochemistry

Gronostajowa 2, 30387 Krakow, Poland

Tel: +48 12 686 25 20

Fax: +48 12 686 27 50

TABLE OF CONTENT

1. Supplementary results S3

1.1. SEM S3

1.2. EDS elemental mapping S3

1.3. TEM analysis S4

1.4. XPS analysis S6

1.5. XRD analysis S6

1.6. Optical properties S7

1.7. Photoelectrochemical properties S7

2. PEC methanol oxidation – literature review S9

3. References S9

1. **Supplementary results**

1.1. SEM

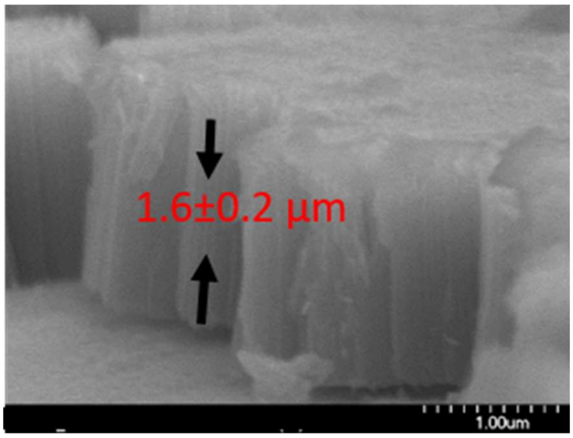

Figure S1. Cross-sectional SEM image of TiO_2 NTs.

1.2. EDS elemental mapping

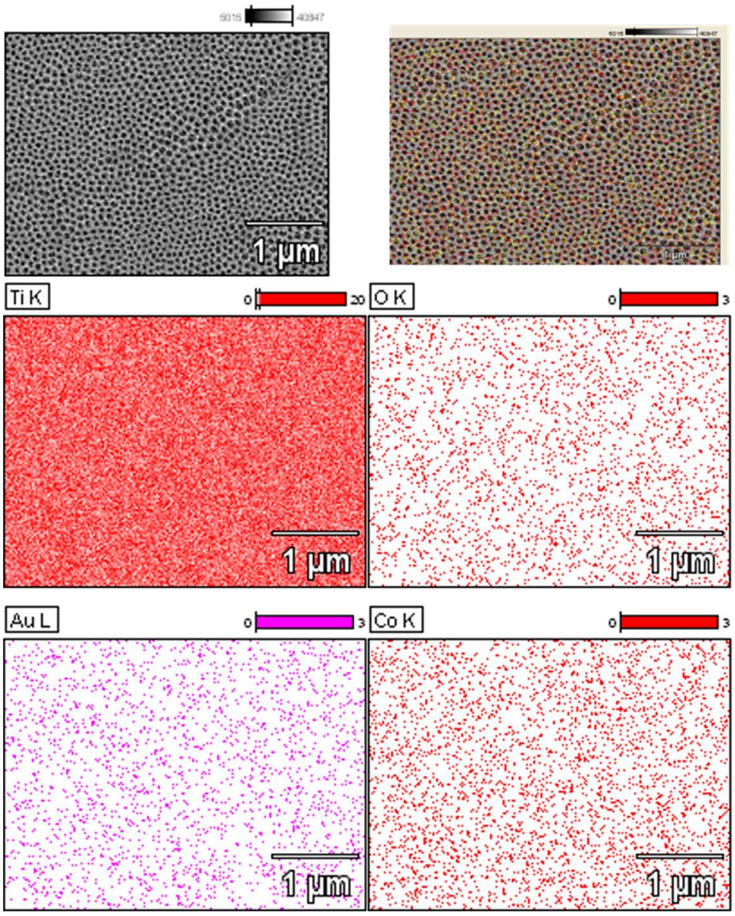

Figure S2. EDS distribution (color elemental mapping) images of TiO_2-Au-CoO_x.

1.3. TEM analysis

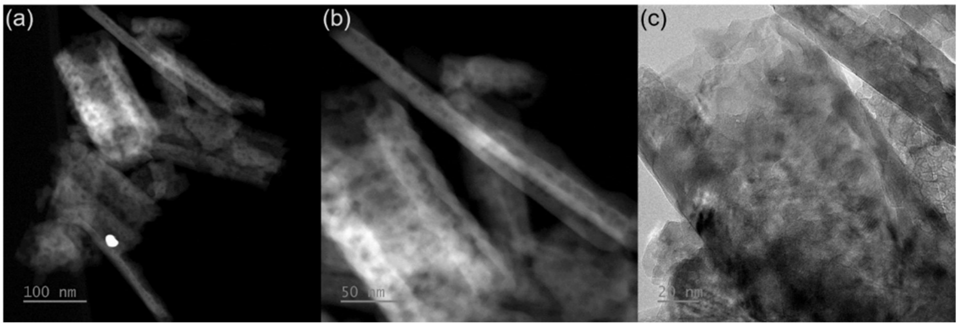

Figure S3. Low and high resolution HAADF-STEM images of TiO_2 NTs.

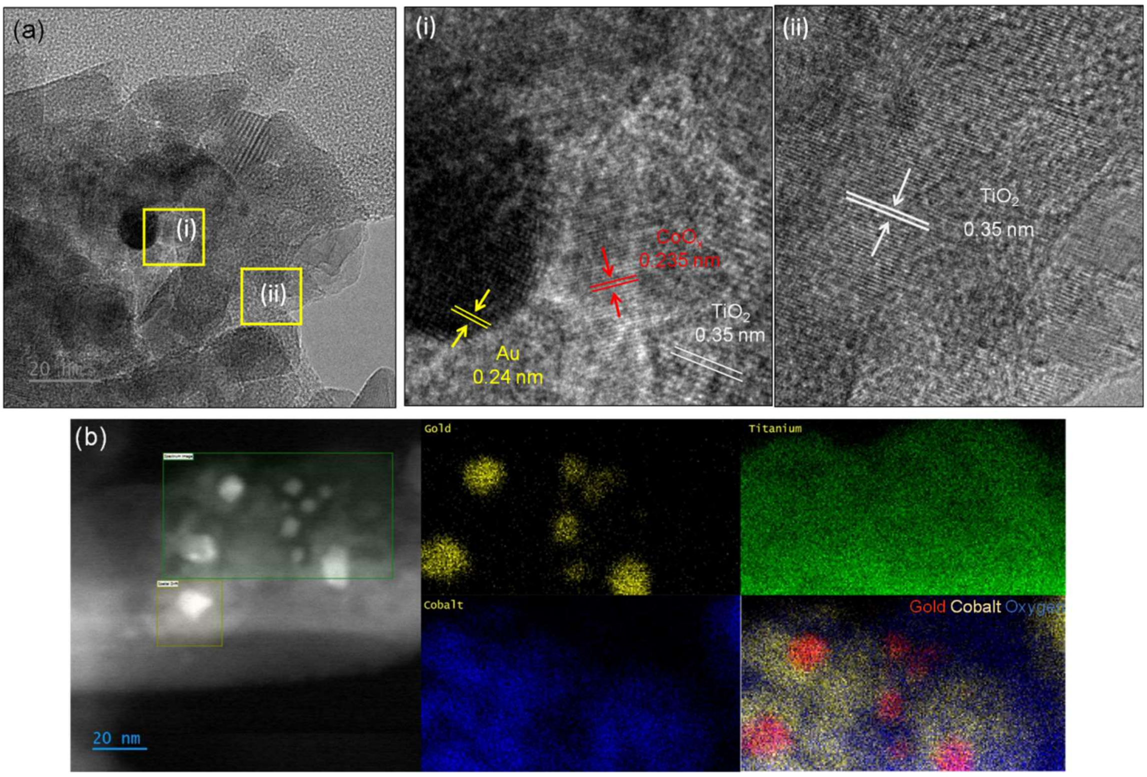

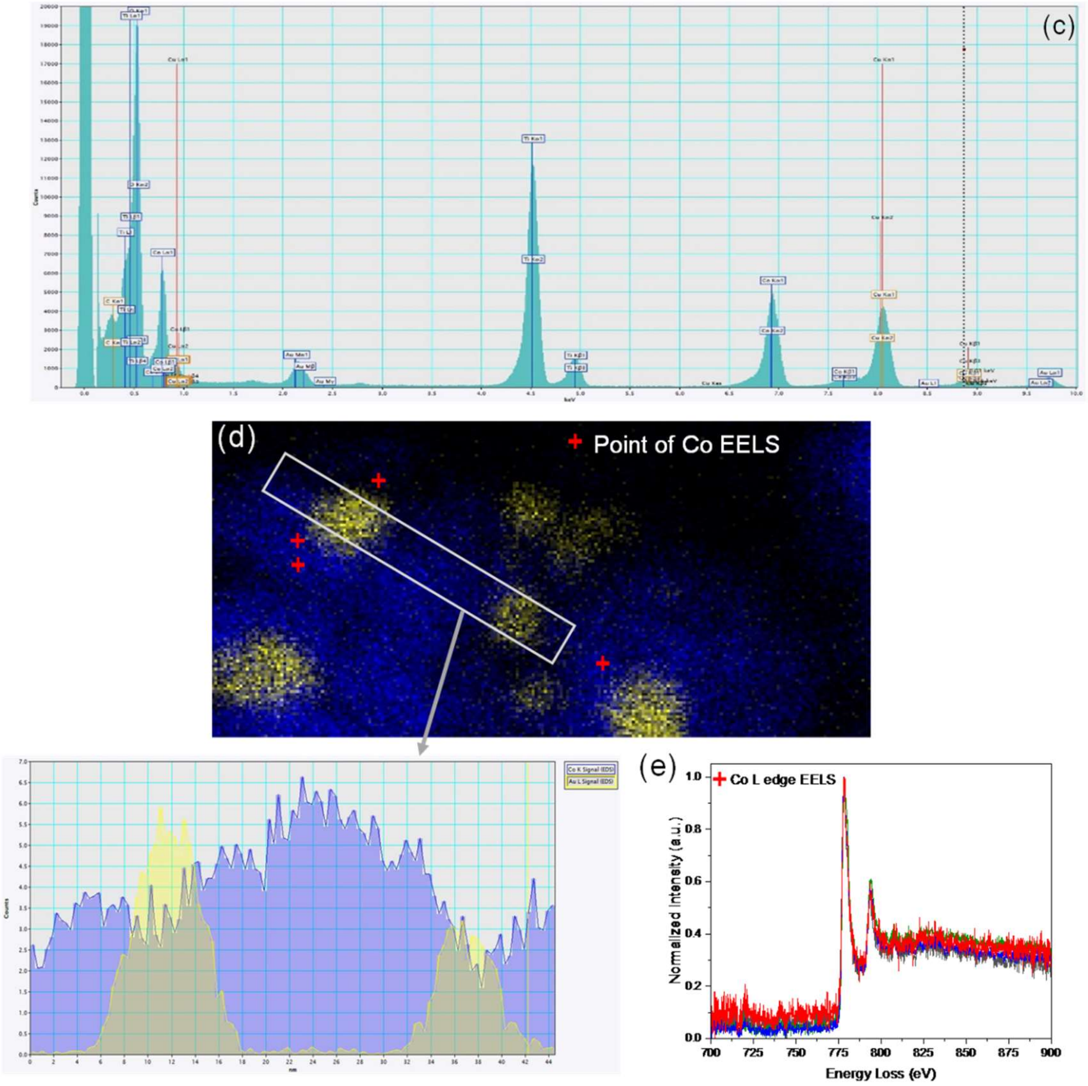

Figure S4. (a) HRTEM image of TiO_2-Au-CoO_x (i and ii marked portion lattice fringe), (b and c) STEM-EDS color mapping of highlighted portion TiO_2-Au-CoO_x, (d) EDS-EELS line scan analysis and (e) average Co L edge EELS of marked (+) points.

1.4. XPS analysis

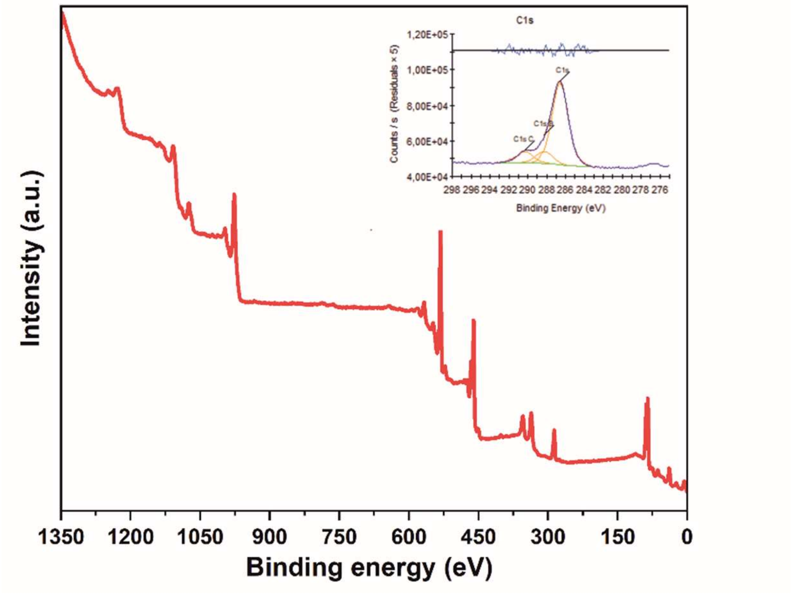

Figure. S5. XPS survey scan and C 1s high resolution spectra.

1.5. XRD analysis

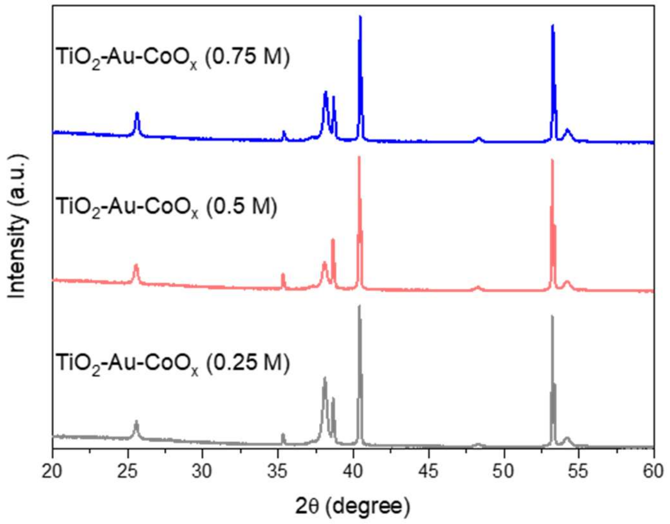

Figure S6.XRD patterns of TiO_2 NTs-Au-CoO_x-0.25 M, 0.5 M, and 0.75 M.

1.6. Optical properties

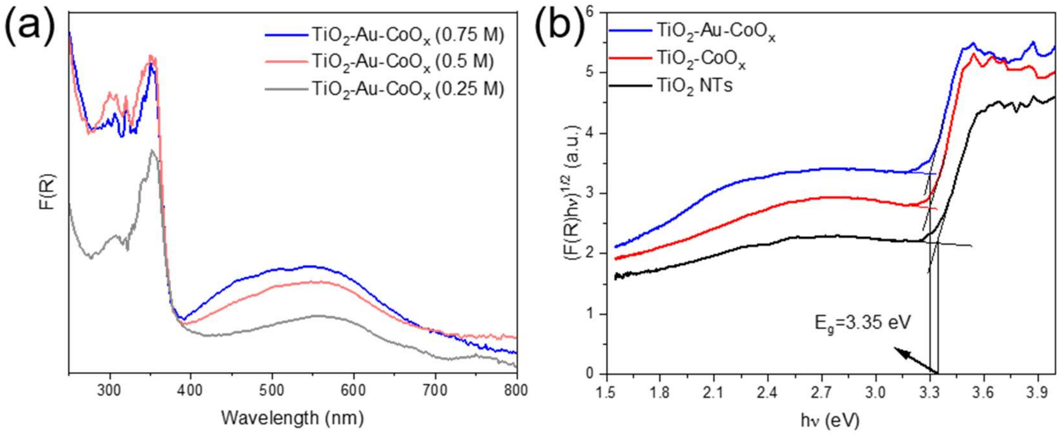

Figure S7.(a) Kubelka Munk plots for TiO_2-Au-CoO_x of different concentrations, and (b) band gap plots of TiO_2 NTs, TiO_2-CoO_x, and TiO_2-Au-CoO_x.

1.7. Photoelectrochemical properties

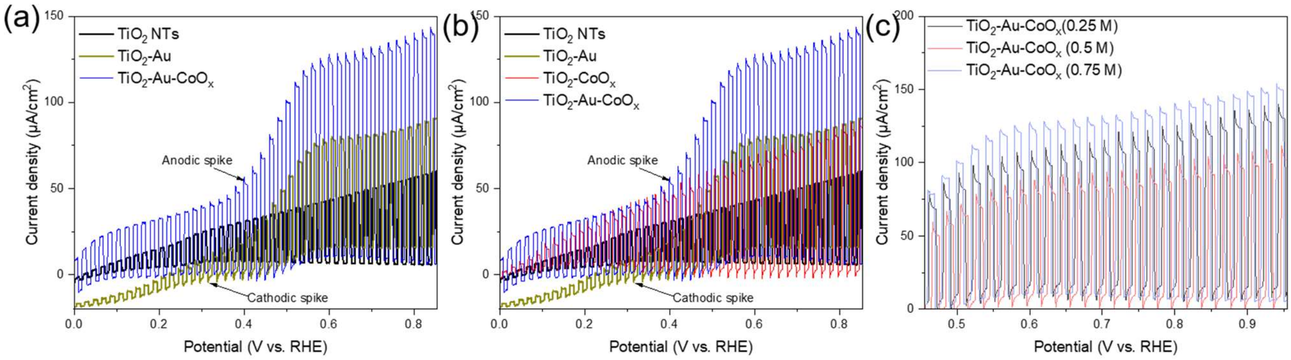

Figure S8. (a and b) Cathodic and anodic spikes of TiO_2 NTs, TiO_2-CoO_x, TiO_2-Au and TiO_2-Au-CoO_x. and (c) different concentrations of CoO_x TiO_2-Au-CoO_x.

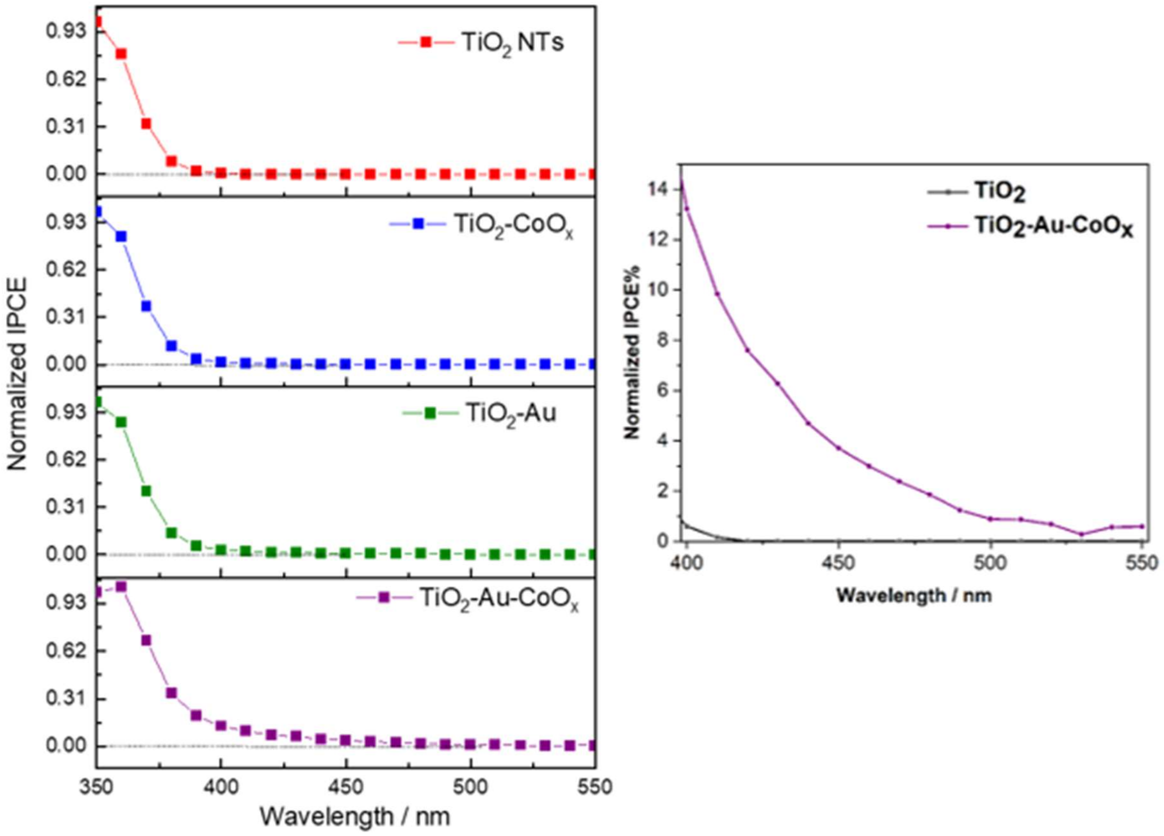

Figure 9. IPCE spectra of tested materials.

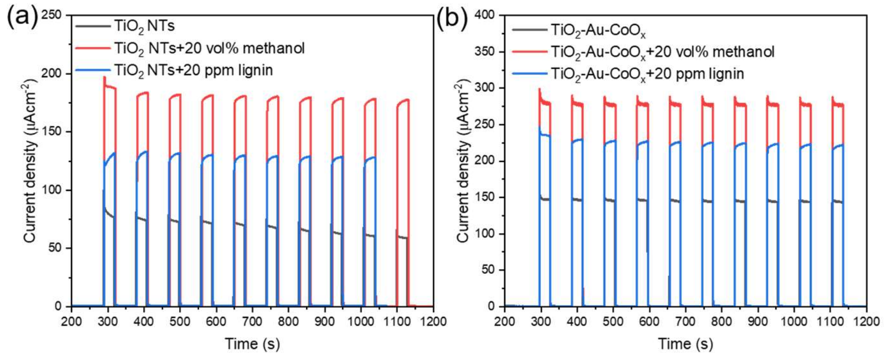

Figure S10.(a) On-off CA plot of TiO_2 and, (b) TiO_2-Au-CoO_x in KNO_3, and KNO_3 containing methanol and lignin.

2. *PEC methanol oxidation – literature review*

Table S1. A comparison between our synthetic system and the latest PEC methanol oxidation activity state of the art.

Photoelectrocatalyst	Electrolyte	Activity (methanol oxidation)	Light	Ref.
Si-doped hematite	0.1 m NaOH + 95% methanol	HCHO≈ 0.37 µmol –FE-95% @1.5 V vs. RHE	AM-1.5	1
$BiVO_4$|Fe:Ni–Bi	0.1 m borate buffer + 0.1 m methanol	HCOOH ≈ 5.7 ± 1.0 µmol/ cm^2h^1 FE-94%@0.55 V vs. RHE	AM-1.5	2
Au-α-Fe_2O_3/ZnO	0.1 m NaOH + 95% methanol	HCHO-FE-79.23% at 0.8 V vs. RHE	Xe-UV-Vis	3
α-Fe_2O_3/$CoFe_2O_4$	0.1 m NaOH + 40% methanol	HCHO ≈ 0.3 mmol/ cm^2h^1 FE-97.8%@0.5 V vs. RHE	Stimulated solar light	4
n-Si/SiO_x/NiFe	1 m KOH + methanol	HCHO-FE-86% at 1.5 V vs. RHE	AM-1.5	5
TiO_2-Au-CoO_x NTs	0.1 M KNO_3 and 0.1 M KOH + 95% methanol	HCHO-1.15 µmol/cm^2h^1 (neutral) and 61.2 µmol/cm^2h^1 (alkaline) @1.5 V vs.RHE	AM-1.5	This work

FE-faradic efficiency

3. References

1. Mesa, C.A., Kafizas, A., Francàs, L., Pendlebury, S.R., Pastor, E., Ma, Y., Le Formal, F., Mayer, M.T., Grätzel, M. and Durrant, J.R., Kinetics of photoelectrochemical oxidation of methanol on hematite photoanodes. *J. Am. Chem. Soci.*, 2017, *139*(33), 11537-11543.

2. Huang, S.C., Cheng, C.C., Lai, Y.H. and Lin, C.Y., Sustainable and selective formic acid production from photoelectrochemical methanol reforming at near-neutral pH using nanoporous nickel-iron oxyhydroxide-borate as the electrocatalyst. *Chem. Eng. J.*, 2020, 395, p.125176.

3. Zheng, B.F., Ouyang, T., Wang, Z., Long, J., Chen, Y. and Liu, Z.Q., Enhanced plasmon-driven photoelectrocatalytic methanol oxidation on Au decorated α-Fe 2 O 3 nanotube arrays. Chem. Commun., 2018, 54(69), pp.9583-9586.

4. Huang, S., Feng, F., Huang, R.T., Ouyang, T., Liu, J. and Liu, Z.Q., Activating C–H bonds by tuning Fe sites and an interfacial effect for enhanced methanol oxidation. *Adv. Mater.*, 2022, 34(50), p.2208438.

5. Li, Y., Ding, C., Li, Y., Zeng, J., Kang, C., Chen, H., Wang, L., He, J. and Li, C., Engineering the inhomogeneity of metal–insulator–semiconductor junctions for photoelectrochemical methanol oxidation. *ACS Appl. Mater. Interfaces*, 2023, 15(51), pp.59403-59412.
